# The Molecular, Genetic, Pathological Basis and Clinical Perspectives of Endometrial Cancer: A Narrative Review

**DOI:** 10.3390/ijms27219511

**Published:** 2026-10-24

**Authors:** Chia-Ling Chang, Jie-Hong Chen, Kuo-Hu Chen

**Affiliations:** 1 Department of Medical Education, Taipei Tzu-Chi Hospital, The Buddhist Tzu-Chi Medical Foundation, Taipei 23142, Taiwan; linda1020257@gmail.com; 2 School of Medicine, College of Medicine, MacKay Medical University, New Taipei City 25245, Taiwan; albertjhc@gmail.com; 3 Department of Obstetrics and Gynecology, Taipei Tzu-Chi Hospital, The Buddhist Tzu-Chi Medical Foundation, New Taipei City 23142, Taiwan; 4 School of Medicine, Tzu-Chi University, Hualien 97004, Taiwan

**Keywords:** endometrial cancer, POLE, mismatch repair, p53, targeted therapy

## Abstract

Endometrial cancer (EC) is the most common gynecologic malignancy in high-income regions, with a rising global incidence driven by population aging and obesity. Management of EC is undergoing a paradigm shift from traditional histopathological classification (Bokhman Types I and II) to a molecularly integrated model. Key driver alterations include mutations in PTEN, PIK3CA, PIK3R1, ARID1A, KRAS, and TP53, alongside focal amplifications of CCNE1 and ERBB2, all of which drive oncogenesis through dysregulation of the PI3K/AKT/mTOR, Wnt/β-catenin, MAPK/ERK, and p53 signaling cascades. Integrated genomic profiling via The Cancer Genome Atlas (TCGA) and practical surrogates like ProMisE classifies EC into four distinct molecular subgroups: POLE-ultramutated (POLEmut), mismatch repair-deficient (MMRd/MSI-H), copy-number low/non-specific molecular profile (NSMP), and copy-number high/p53-abnormal (p53abn). These subgroups display unique biological features, tumor microenvironments, and prognostic trajectories, and have been formally incorporated into the FIGO 2023 staging system. Crucially, molecular classification directly informs biomarker-driven systemic management. MMRd may predict profound responses to immune checkpoint inhibitors (e.g., pembrolizumab, dostarlimab) in both frontline and recurrent settings. For MMR-proficient disease, combination strategies such as anti-angiogenic/immunotherapy (lenvatinib plus pembrolizumab), hormone/mTOR or CDK4/6 inhibitor combinations, and nuclear export inhibitors (selinexor in *TP53* wild-type) seem to offer substantial benefit. Furthermore, novel modalities including HER2-targeted antibody-drug conjugates (trastuzumab deruxtecan), PARP inhibitors, and G2/M checkpoint inhibitors (adavosertib) are actively expanding the therapeutic armamentarium. Integrating multi-omics techniques, artificial intelligence, and novel systemic therapies into clinical workflows emerge to refine risk stratification, avoid over- or undertreatment, and establish a new standard of precision oncology in endometrial cancer.

## 1. Introduction

Endometrial cancer (EC) is the most common gynecologic malignancy in developed countries. In the United States, it ranks as the fourth most frequent cancer among women, with an estimated 68,270 new cases and 14,450 deaths projected for 2026 [1]. Globally, EC is the sixth most common cancer in women, with approximately 420,000 new cases and over 97,000 deaths each year [2].

The burden of EC is disproportionately higher in high-income regions, including North America, Europe, and Oceania, where aging populations and rising obesity rates contribute to an increasing incidence [2,3]. In the United States, the incidence has risen by approximately 1.8% to 2.6% annually since the mid-2000s [1]. In contrast, although the incidence rates remain lower in developing regions such as Africa, Asia, and Latin America, mortality rates are disproportionately higher, largely due to limited access to early diagnosis and effective treatment [2,3]. Overall, EC represents a growing global public health concern and highlights persistent disparities in healthcare access and outcomes.

EC primarily affects postmenopausal women, with a median age at diagnosis of 61 years, and most cases occur between ages 45 and 74 [4]. Several factors contribute to the rising incidence of endometrial cancer (EC), including increased life expectancy, the growing prevalence of obesity, and prolonged exposure to unopposed estrogen, which is commonly associated with nulliparity, infertility (e.g., polycystic ovary syndrome), and tamoxifen use. Obesity is the most prominent risk factor, accounting for up to 57% of EC cases in high-incidence countries [5]. This effect is primarily mediated by increased estrogen production through aromatization of androgens in adipose tissue, promoting endometrial hyperplasia and subsequent malignant transformation. Additionally, other components of metabolic syndrome—such as hypertension, hyperinsulinemia, and type 2 diabetes—are also associated with an increased risk of EC [6].

For decades, management of EC was guided by the dualistic model proposed by Bokhman in 1983. Type I (endometrioid) represents 60–80% of cases, typically estrogen-dependent, associated with obesity, presenting at an early stage with a relatively favorable prognosis [6,7]. Type II (non-endometrioid) EC includes serous, clear cell, and carcinosarcoma; these are generally estrogen-independent, occuring in older/thinner women, and are highly aggressive with a high risk of relapse and poor prognosis [7,8]. While the traditional model provides a useful foundation, it is limited by poor interobserver reproducibility in histologic grading and subtyping, particularly in high-grade tumors [9,10]. Such variability may lead to overtreatment or undertreatment in a proportion of patients [10].

The management of EC is currently in a transition period from a purely histopathological approach to one integrated with molecular phenotypes and genomic profiling [5,6]. In 2013, The Cancer Genome Atlas (TCGA) published a disruptive genomic analysis that redefined EC into four distinct molecular subgroups based on mutational burden and copy number alterations: polymerase epsilon (POLE)-ultramutated, microsatellite instability hypermutated (MSI-H), copy-number low (CNL), and copy-number high (CNH) [7,11]. This shift allows for a more objective, biologically relevant categorization that better predicts clinical behavior [6,11]. This review approaches EC from a mechanistic perspective. Although molecular classification guides cancer prognosis and treatment, it does not explain why tumors within the same class or stage respond differently to current therapy. We therefore examine the signaling pathways driving EC, and link them to therapeutic response, resistance, and combination strategies. Molecular classification serves as the framework, with histopathology, targeted therapy, immunotherapy and future directions discussed accordingly.

## 2. Methods of Literature Review

**Figure 1:**
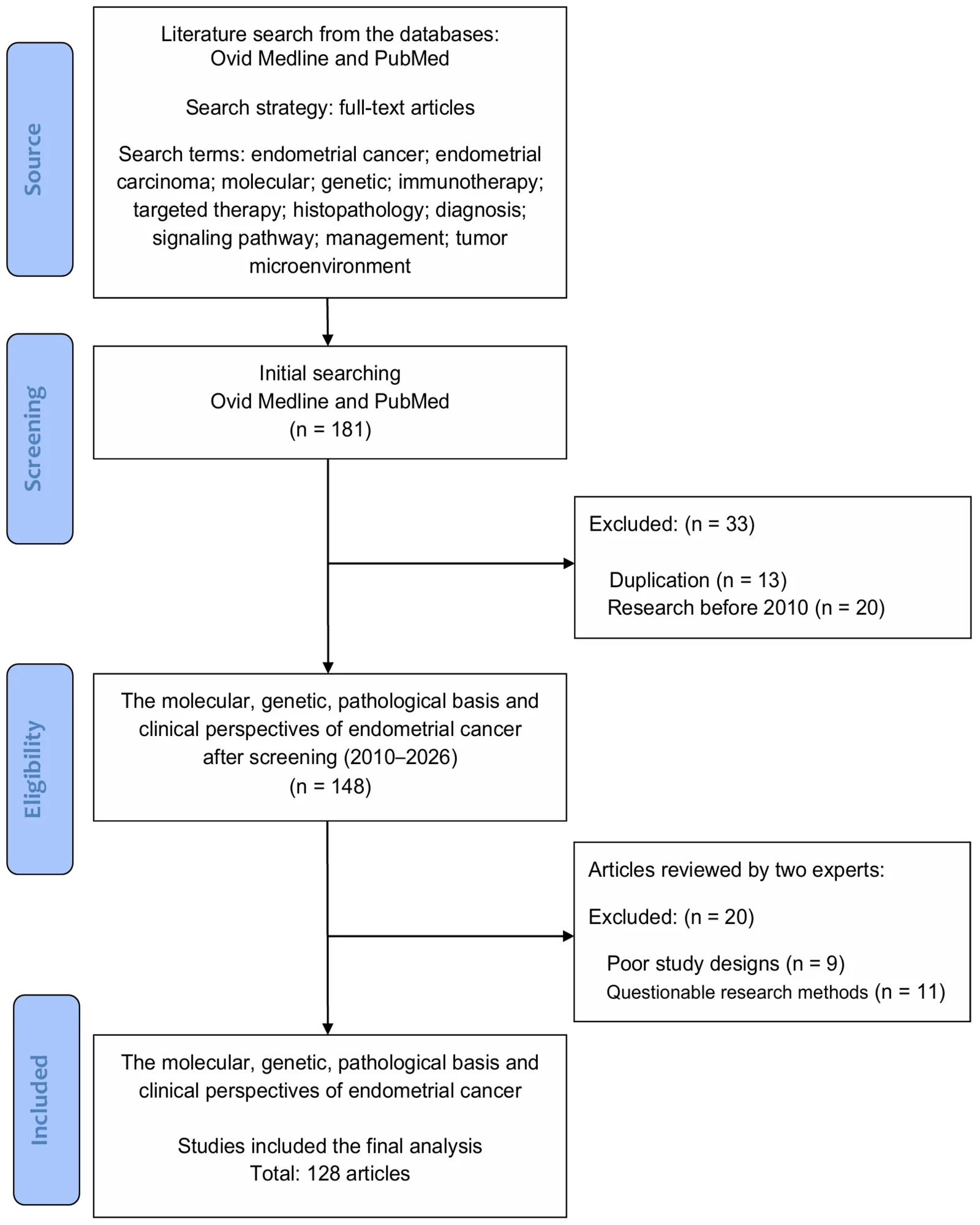
The flowchart of source, database searching, screening, exclusion and the inclusion of the references that we collected from the literature.

The literature was searched to retrieve basic and clinical research that investigated the underlying molecular and cellular mechanisms of EC, along with the treatment. Figure 1 illustrates the flowchart of database searching, screening, and the inclusion of the references that we collected from the literature. A comprehensive literature search was conducted to identify both basic and clinical studies investigating the molecular, genetic, and pathological basis, signaling pathways, diagnostic approaches, and clinical management of EC. In the current review, relevant articles were retrieved from the Ovid Medline and PubMed databases using the following search terms: “endometrial cancer”, “endometrial carcinoma”, “molecular”, “genetic”, “immunotherapy”, “targeted therapy”, “histopathology”, “diagnosis”, “signaling pathway”, “management”, and “tumor microenvironment”. For screening and selection in the second stage, only full-text articles published in English were considered for further evaluation.

In the next stage, duplicated articles were removed after the initial search. The articles published before 2010 were also excluded to ensure the novelty of this review. From a total of 181 articles identified initially in the screening process, 148 articles (2010–2026) met the criteria for further inclusion.

Hereafter, two experts in the field independently inspected the contents of the articles, including research designs and materials, and identified the eligible basic and clinical research for inclusion. The articles with poor study design, questionable methodology (e.g., inappropriate patient selection and grouping), or unclear outcomes (e.g., no clear definition of disease or treatment endpoint) were excluded in this stage. The discrepancies between two experts were resolved through mutual discussion to reach a consensus. According to the inclusion criteria (full-text articles; search terms; year 2010–2026) and exclusion criteria (duplicated articles; articles published before 2010; poor research designs and questionable research methods), all eligible articles were included in this review using the search terms and strategies (source, screening of the potential articles, selection of studies, and final inclusion). Finally, a total of 128 articles were included for this review from 181 articles solicited in the initial search.

## 3. Histopathology

### 3.1. WHO Classification

The World Health Organization (WHO) classification 5th edition (2020) provides the current comprehensive framework for categorizing EC, distinguishing tumors based on their distinct histopathological features and biological behavior. While for decades EC was dichotomized into the Bokhman Type I and Type II model, the current standard recognizes a more heterogeneous spectrum of epithelial malignancies [5,6,12]. The major histological types include endometrioid, serous, clear cell, undifferentiated and dedifferentiated carcinoma, and carcinosarcoma [13].

Endometrioid endometrial carcinoma (EEC) is the most common subtype, accounting for over 80% of cases. It is typically estrogen-dependent and is morphologically characterized by glandular or villoglandular architecture lined by columnar cells with pseudostratified nuclei [13]. EEC is traditionally graded from 1 to 3 based on the proportion of solid, non-squamous growth: grade 1 (≤5%), grade 2 (6–50%), and grade 3 (>50%) [14]. Current practices are moving toward a two-tier grading system, grouping grades 1 and 2 as low-grade and grade 3 as high-grade [6]. Low-grade tumors generally have a favorable prognosis, whereas high-grade tumors are more aggressive and are often associated with mismatch repair deficiency or POLE mutations [15]. Tumors commonly express estrogen receptor (ER) and progesterone receptor (PR), with stronger and more diffuse staining observed in low-grade tumors compared to high-grade tumors [5,13,14].

Serous carcinoma (SEC) accounts for approximately 10% of endometrial cancer cases but is responsible for nearly 40% of EC-related deaths, reflecting its highly aggressive nature. Histologically, it is characterized by papillary or glandular architecture, marked nuclear pleomorphism, and high mitotic activity. A key molecular hallmark is the near-universal presence of TP53 mutations, leading to aberrant p53 expression, typically seen as either diffuse overexpression or a complete “null” pattern [5,12,16].

Clear cell carcinoma (CCC) is a rare but aggressive subtype, accounting for less than 5% of endometrial cancer cases. It is characterized by tubulocystic, papillary, or solid growth patterns with distinctive clear cytoplasm and hobnail cells [5]. These tumors typically lack estrogen and progesterone receptor expression and are often resistant to chemotherapy. Compared with low-grade endometrioid carcinoma, CCC is associated with a poorer prognosis [8,12].

Carcinosarcoma (CS), previously known as malignant mixed Müllerian tumor, is a rare biphasic carcinoma accounting for less than 5% of all ECs [17,18]. It consists of both malignant epithelial (carcinomatous) and mesenchymal (sarcomatous) components and is further classified based on whether the sarcomatous element is native to uterine tissue (homologous) or non-native (heterologous) [5,17,19]. Although historically categorized as a sarcoma, it is now recognized as a metaplastic carcinoma arising from a single precursor lesion, with the sarcomatous component developing through metaplastic differentiation [5]. Its patterns of recurrence and metastasis closely resemble those of endometrial carcinoma, and most cases harbor TP53 mutations, contributing to an aggressive clinical course and very poor prognosis [5,11,17].

Undifferentiated and dedifferentiated carcinoma (UDC/DDC) are rare, highly aggressive, and often underrecognized endometrial cancer subtypes [12]. Undifferentiated carcinoma is characterized by discohesive sheets of monomorphic cells with minimal or no evidence of epithelial differentiation. In contrast, dedifferentiated carcinoma is defined by the coexistence of a low-grade endometrioid carcinoma component alongside an undifferentiated component [5,20]. These tumors frequently exhibit loss of SWI/SNF complex proteins and are commonly associated with a high rate of mismatch repair (MMR) deficiency [5,14].

The histopathological characteristics of major endometrial cancer subtypes are shown in Table 1.

### 3.2. Precursor Lesions

The pathogenesis of EC follows two principal pathways; each is associated with distinct precursor lesions and molecular mechanisms. The first pathway, typically leading to endometrioid endometrial carcinoma (EEC), is estrogen-dependent and arises in the setting of prolonged unopposed estrogen exposure [5]. Precursor lesions include endometrial hyperplasia and endometrial intraepithelial neoplasia (EIN), the latter representing a clonal proliferation of atypical glandular cells with an increased gland-to-stroma ratio compared to surrounding tissue [5,16,21]. EIN has largely replaced the older classification of atypical hyperplasia and is associated with a significantly increased risk of malignant transformation, with studies showing that up to 43% of patients may harbor concurrent carcinoma at diagnosis [16,18,21]. The progression from normal endometrium to EIN and eventually carcinoma is driven by the accumulation of somatic mutations, most commonly involving PTEN, as well as PIK3CA, KRAS, and ARID1A, supporting a model of stepwise clonal evolution [3,21].

In contrast, the second pathway leads to serous carcinoma and is estrogen independent. Its precursor lesion, endometrial intraepithelial carcinoma (EIC), also referred to as serous endometrial intraepithelial carcinoma (SEIC), typically arises in atrophic endometrium or on the surface of endometrial polyps. This pathway is characterized by early TP53 mutations [5,16]. Together, these two distinct pathways highlight the divergent biological mechanisms underlying EC and underscore the importance of integrating hormonal, histological, and molecular features in understanding disease development.

## 4. Genetic Alterations and Genomic Landscape

The understanding of EC has evolved from a traditional dualistic histopathological model to a comprehensive genomic characterization [22]. Research, notably by TCGA, has revealed that EC is a heterogeneous disease driven by a wide array of somatic mutations, DNA repair defects, and copy number alterations. These genetic events occur across a spectrum of mutational burdens, ranging from nearly diploid tumors with few alterations to ultramutated phenotypes with over 100 mutations per megabase [5]. Table 2 lists major genetic alterations and genomic features in endometrial cancer.

### 4.1. Driver Mutations: PTEN, PIK3CA, PIK3R1, TP53, ARID1A, KRAS

The pathogenesis of EC is often driven by the stepwise accumulation of mutations in key driver genes that regulate cell survival, proliferation, chromatin remodeling, and cell cycle control [3]. These alterations often occur early in tumorigenesis, promoting the progression from normal endometrial epithelium through precursor lesions to invasive carcinoma [15].

#### 4.1.1. PTEN

PTEN is the most commonly altered tumor suppressor gene in EC, occurring in approximately 40–80% of cases, particularly in the endometrioid subtype [3,25]. Located on chromosome 10q23, PTEN encodes a phosphatase that negatively regulates the PI3K/AKT signaling pathway by converting PIP3 to PIP2 [3]. Loss of PTEN function—through mutation or promoter hypermethylation—leads to constitutive pathway activation, resulting in increased cell proliferation, survival, and migration. These alterations proceed early in tumorigenesis and are frequently observed in precursor lesions such as atypical endometrial hyperplasia (AEH) [3,15,26].

#### 4.1.2. PIK3CA and PIK3R1

PIK3CA and PIK3R1 encode key subunits of the phosphatidylinositol-3-kinase (PI3K) complex and play central roles in activation of the PI3K/AKT/mTOR signaling pathway [23]. PIK3CA encodes the p110α catalytic subunit and is mutated in approximately 40–50% of ECs, with gain-of-function mutations most commonly occurring in the helical (exon 9) and kinase (exon 20) domains, leading to increased PI3K activity [3,27].

#### 4.1.3. TP53

TP53, known as the “guardian of the genome”, is a critical tumor suppressor that regulates the cell cycle, DNA repair, and apoptosis [7]. It is frequently altered in high-grade and aggressive endometrial cancer subtypes, with mutations identified in over 90% of serous carcinomas and carcinosarcomas [15]. These alterations lead to genomic instability, including aneuploidy and extensive copy number changes [8,22]. In serous endometrial cancer, TP53 mutations are considered to occur early, potentially initiating events in carcinogenesis and are often present in precursor lesions such as SEIC [5,18].

#### 4.1.4. ARID1A

AT-rich interactive domain 1A (ARID1A) encodes a key subunit of the SWI/SNF chromatin remodeling complex and plays an essential role in regulating gene expression and maintaining genomic stability [28]. Mutations in ARID1A are identified in approximately 30–50% of ECs and typically result in loss of function through frameshift or nonsense mutations [3]. This loss disrupts chromatin remodeling, alters histone acetylation, and leads to silencing of tumor suppressor genes [29].

#### 4.1.5. KRAS

Mutations in the KRAS oncogene are observed in approximately 10–30% of EC cases and result in activation of the MAPK/ERK signaling pathway, promoting cell proliferation and survival [28]. These mutations are often associated with specific morphological features, such as mucinous differentiation [14]. KRAS alterations are frequently identified in areas of endometrial hyperplasia, suggesting a role in early tumorigenesis [28]. In addition, KRAS mutations may contribute to activation of the WNT/β-catenin pathway through alternative mechanisms [15].

### 4.2. DNA Repair Defects: Mismatch Repair (MMR) Genes and Microsatellite Instability (MSI)

Approximately 25–30% of ECs exhibit DNA mismatch repair deficiency (MMRd). The MMR system plays a critical role in maintaining genomic stability by correcting base-pairing errors and small insertions or deletions that occur during DNA replication [8,30]. This system involves four key proteins—MLH1, MSH2, MSH6, and PMS2—which function as heterodimers: MSH2–MSH6 recognizes mismatched bases, while MLH1–PMS2 mediates the repair process [7,8,18]. Microsatellites are short, repetitive DNA sequences prone to replication errors. Loss of MMR function leads to accumulation of replication errors in microsatellites, resulting in microsatellite instability (MSI) [5,31]. This results in a hypermutated phenotype, with MSI-high tumors exhibiting a tumor mutational burden (TMB) approximately ten times higher than mismatch repair–proficient (MMRp) tumors [8,18].

MMRd in EC arises through three main mechanisms. The most common is sporadic MLH1 promoter hypermethylation (70–80%), leading to loss of MLH1 and PMS2 expression [5,7,11]. A smaller proportion, about 3% of all EC cases, are associated with Lynch syndrome, caused by germline mutations in MMR genes, most commonly MSH2, MSH6, or MLH1 [5,31]. For 50% of women with Lynch syndrome, EC is their sentinel malignancy [8]. In addition, approximately 10–20% of MMRd cases result from double somatic alterations in MMR genes, termed “Lynch-like” syndrome [5,11,21].

### 4.3. Ultramutation and POLE Mutations

A distinct and clinically significant subset of ECs, comprising approximately 7–10% of cases, is defined by an ultramutated phenotype [5,28]. This group represents the extreme end of the mutational spectrum, with a TMB that typically exceeds 100 mutations per megabase (mut/Mb)—about 100 times higher than the average across all EC subtypes [5,6,32].

This phenotype is driven by somatic mutations in the exonuclease domain (EDM) of the POLE gene [32]. POLE encodes the subunit of DNA polymerase epsilon, a key enzyme responsible for high-fidelity leading-strand DNA replication in eukaryotic cells. The EDM is essential for proofreading, a process where mismatched bases are identified and replaced during DNA synthesis [7,8]. However, pathogenic mutations in this domain lead to the massive accumulation of single-nucleotide substitutions [30]. Most pathogenic POLE variants are localized within the EDM, particularly in exons 9, 11, 13, and 14 [8]. To date, 11 missense mutations have been identified as key hotspots driving the ultramutated phenotype [32]. Among these, five variants—P286R, V411L, S297F, A456P, and S459F—account for approximately 95% of all POLE-mutant cases [7,32].

The ultramutated genome is characterized by a unique mutational signature, which features a markedly increased frequency of C>A transversions, often accounting for more than 20% of all genomic alterations [15,32]. Despite the extreme number of mutations, these tumors are generally microsatellite stable (MSS), although rare “dual classifier” tumors harboring both POLE mutations and MMRd can occur [7,15]. The extensive mutational load leads to an exceptionally high frequency of mutations in other critical driver genes. In this subtype, mutations in PTEN are found in approximately 94% of cases, frequently co-occurring with mutations in FBXW7 (82%), PIK3CA (71%), ARID1A (66%), and PIK3R1 (65%) [15,32].

### 4.4. Copy Number Alterations: CCNE1, ERBB2

Beyond point mutations, EC is characterized by varying degrees of somatic copy number alterations (SCNAs), including broad or focal chromosomal gains and losses [15]. Widespread SCNAs are most prevalent in high-grade, aggressive tumors that typically lack MSI or POLE mutations [5]. Significant focal amplifications often involve key oncogenes that drive cell cycle progression and proliferation, most notably CCNE1 and ERBB2 [18].

#### 4.4.1. CCNE1

CCNE1 gene, located on chromosome 19q12, encodes Cyclin E1, a key regulatory protein that facilitates the transition from the G1 to the S phase of the cell cycle [5,24]. Focal amplification of CCNE1 leads to overexpression of Cyclin E1, resulting in marked cell cycle deregulation and increased replication stress, a hallmark of aggressive tumor biology [18,24]. Genomically, CCNE1 amplification is frequently observed in tumors with extensive SCNAs, particularly in uterine serous carcinomas and high-grade endometrioid carcinomas [18,33]. These amplifications are thought to contribute to chemoresistance, further complicating the poor clinical outcomes of these aggressive subtypes [34].

#### 4.4.2. ERBB2

The ERBB2 oncogene, located on chromosome 17q12, encodes human epidermal growth factor receptor 2 (HER2), a transmembrane tyrosine kinase receptor involved in the regulation of cell growth, differentiation, and survival [25,28]. Amplification of ERBB2 is observed in approximately 25% of copy-number high (serous-like) endometrial cancers and is also prevalent in uterine carcinosarcomas, with reported rates ranging from 17% to 33% [6,22,25].

A defining feature of HER2-amplified endometrial cancer is its strong association with TP53 mutations, which are present in 94% to 97% of cases. This co-occurrence suggests that HER2 amplification frequently arises in the context of p53 dysfunction and may represent a later event in tumor progression [25]. In addition, intratumoral heterogeneity of HER2 expression and gene amplification is common (approximately 74%), characterized by variable staining intensity and different levels of gene amplification across tumor cell populations [35].

### 4.5. Other Relevant Alterations

Additional genetic events contribute to the complex genomic landscape of EC. Mutations in CTNNB1 (β-catenin) occur in approximately 30–50% of tumors with low copy number alterations and are associated with activation of the WNT signaling pathway [7,36]. Although often identified in low-grade and early-stage tumors, CTNNB1 mutations are independent predictors of poorer recurrence-free survival and an increased risk of distant metastasis [11,14,37].

Mutations in PPP2R1A and FBXW7 are also frequently observed, particularly in high-grade ECs, especially within the p53-abnormal (copy-number high) subtype [15,38]. In addition, overexpression of L1 cell adhesion molecule (L1CAM) is a strong independent predictor of distant recurrence and decreased survival [6,11]. It is most prevalent in p53-abnormal tumors but also serves as an important marker for identifying high-risk patients within the NSMP group [6,37].

## 5. Molecular Classification of Endometrial Cancer

### 5.1. TCGA Classification

The molecular landscape of endometrial cancer (EC) was fundamentally redefined in 2013 by The Cancer Genome Atlas (TCGA), which applied integrated genomic, transcriptomic, and proteomic analyses to move beyond the traditional dualistic histopathologic model [5,6,16]. By analyzing 373 cases, the TCGA identified four distinct molecular subgroups based on their mutational burden and copy-number alteration [5]. This classification has demonstrated improved reproducibility and prognostic accuracy compared with conventional histotyping [11]. Afterwards, the 2023 FIGO staging system for endometrial cancer formally incorporates TCGA molecular classification alongside traditional anatomic findings to guide prognosis and treatment, including four molecular subtypes: POLEmut (POLE ultramutated), MMRd (mismatch repair-deficient/microsatellite instable), NSMP (non-specific molecular profile) and p53abn (p53 abnormal) [39]. Table 3 lists molecular classification and features of endometrial cancer according to TCGA and ProMisE.

#### 5.1.1. POLE Ultramutated

The POLE ultramutated subgroup accounts for approximately 7–10% of all ECs [5,28]. It is characterized by pathogenic mutations in the exonuclease domain of the POLE gene, result in high TMB [5,6,32]. The extremely high mutation burden leads to the generation of numerous neoantigens, thereby enhancing immune recognition of tumor cells. Clinically, these tumors are often high-grade (FIGO grade 3) and occur in younger women with lower BMI; however, they are associated with an excellent prognosis and minimal recurrence risk [17,21].

#### 5.1.2. MSI Hypermutated

The MSI hypermutated subgroup, representing about 28–30% of cases, is characterized by MMRd [5,9,33]. This deficiency, often resulting from MLH1 promoter hypermethylation or germline mutations (Lynch Syndrome), leads to a high rate of insertions and deletions (indels) and a TMB roughly 10-fold higher than the general background (10–100 mutations/Mb) [3,16,40]. These tumors are predominantly endometrioid, frequently present with prominent tumor-infiltrating lymphocytes (TILs), and exhibit an intermediate prognosis [14,18,39].

#### 5.1.3. Copy-Number Low (CNL)

The CNL subgroup constitutes the largest and most heterogeneous molecular subtype, encompassing 38–40% of ECs [5,33]. These tumors exhibit low mutation rates (<10 mutations/Mb) and relatively low SCNA [40]. They are primarily low-grade endometrioid carcinomas that are hormone receptor positive [3]. While generally associated with a favorable outcome, a subset harboring CTNNB1 exon 3 mutations may demonstrate more aggressive behavior [37].

#### 5.1.4. Copy-Number High (CNH)

In contrast, the CNH subgroup, accounting for approximately 26% of cases, carries the poorest prognosis [33]. It is marked by extensive SCNAs, and near-universal (approx. 90–95%) pathogenic TP53 mutations [16,19]. Although this group primarily comprises serous carcinomas, it also includes about 25% of high-grade endometrioid carcinomas and most carcinosarcomas [39]. These tumors are characterized by high cytonuclear atypia and an aggressive clinical course, often responsible for a disproportionate amount of EC mortality [3,14].

Overall, the TCGA classification has transitioned EC management from a purely morphological assessment to a biology-driven risk stratification system [41]. This genomic framework provided the basis for the development of practical clinical surrogates like ProMisE and led to the formal integration of molecular parameters into the FIGO 2023 staging system [39].

### 5.2. ProMisE

The Proactive Molecular Risk Classifier for Endometrial Cancer (ProMisE) is developed to translate the complex genomic findings of the TCGA into a practical and cost-effective clinical tool [9,16]. While the original TCGA classification relies on expensive methodologies like whole-genome and exome sequencing, ProMisE utilizes widely available immunohistochemistry (IHC) and targeted sequencing of a single gene (POLE) to categorize tumors into four analogous subgroups [28]. This classifier has been extensively validated for its feasibility and strong prognostic performance, demonstrating high reproducibility across diagnostic specimens such as biopsies and curettages [10].

The ProMisE categorization follows a hierarchical, stepwise decision tree to ensure consistent and mutually exclusive categorization of ECs [11]. In the original algorithm, MMR IHC was performed first; however, in current practice, MMR IHC, POLE sequencing, and p53 IHC are ideally performed in parallel and interpreted hierarchically, with POLE status assessed first [10,39]. MMR IHC assesses the expression of four key proteins: MLH1, MSH2, MSH6, and PMS2 [8]. Loss of nuclear expression in any of these proteins identifies the MMRd subgroup in POLE wild-type tumors, corresponding to the TCGA MSI-hypermutated category [5,11].

Targeted sequencing of the exonuclease domain of the POLE gene should be performed regardless of MMR status, typically using Sanger sequencing or next-generation sequencing (NGS) [10,42]. The detection of one of the 11 well-characterized pathogenic hotspot mutations, such as P286R, V411L, or S297F, classifies the tumor as POLE-mutant (POLEmut), even in the presence of concurrent MMRd or p53abn [8,9,30]. Restricting POLE testing to MMR-proficient tumors would therefore misclassify POLEmut–MMRd tumors as MMRd [30].

In tumors that are both MMR-proficient and POLE wild-type, p53 IHC is used as a near-perfect surrogate for TP53 mutation status [6,8,9]. An abnormal staining pattern—such as complete absence (null), diffuse strong overexpression, or rare cytoplasmic staining—identifies the p53-abnormal (p53abn) subgroup, which corresponds to the TCGA copy-number high category [14,21].

Tumors that lack MMRd, POLE mutations, and p53 abnormalities are classified as having no specific molecular profile (NSMP), also referred to as p53 wild type (p53wt) [9,18]. This subgroup corresponds to the TCGA copy-number low category and represents the largest and most heterogeneous subset of EC [11].

The TCGA genomic classification and the ProMisE classification system are closely related but are not fully equivalent or interchangeable. ProMisE has clear prognostic value; however, it does not provide a one-to-one correspondence with the TCGA genomic groups. For example, MMR IHC and MSI testing may yield discordant results, and equivocal or heterogeneous IHC findings may require confirmation by MSI-PCR or TP53 sequencing. In addition, NSMP is defined by exclusion and represents a biologically heterogeneous group. Therefore, in this review, the terms “MSI-hypermutated” and “copy-number high/low” are used specifically for the TCGA genomic classification, whereas “MMRd”, “p53abn”, and “NSMP” refer to the molecular classification used in clinical practice.

ProMisE has proven to be an independent prognostic marker that outperforms traditional histological risk stratification [9]. Clinical validation shows that POLEmut tumors have an excellent prognosis, MMRd and NSMP tumors have intermediate outcomes, and p53abn tumors portend the poorest prognosis [41]. Furthermore, approximately 3–6% of cases are identified as “multiple classifiers”, possessing more than one molecular feature (e.g., both POLEmut and p53abn) [43]. In such instances, current guidelines follow a hierarchical logic where POLE mutations override other features due to their dominant favorable outcome, followed by MMR deficiency, while p53 abnormalities are typically considered secondary events in hyper or ultramutated backgrounds [39,43].

The ProMisE molecular subtypes can also be identified in uterine carcinosarcoma (UCS) and provide prognostic stratification. However, their prognostic implications in UCS are not entirely equivalent to those in endometrioid EC or serous carcinoma.

POLEmut tumors account for approximately 2.7–7% of UCS cases and are associated with an excellent prognosis. Several cohort studies have reported virtually no recurrence or disease-related mortality in this subgroup, with outcomes comparable to those of POLEmut EEC. MMRd tumors comprise approximately 7–27% of cases and generally have more favorable outcomes than p53abn or NSMP tumors. Although progression-free survival (PFS) appears comparable to that of EEC within the same molecular subgroup, overall survival (OS) remains poorer, and the precise prognostic significance of this subtype requires further investigation [44,45,46].

The p53abn/TP53-mutated subtype is the most prevalent, accounting for up to approximately 78–85% of UCS cases in some studies and is associated with poor outcomes. Both PFS and OS are significantly worse than those of endometrioid or serous carcinomas within the corresponding molecular subgroup. Similarly, NSMP UCS has an unfavorable prognosis, potentially even worse than that of p53abn endometrioid or serous carcinoma, indicating that NSMP should not be regarded as a low-risk subgroup in UCS [44,45,46].

Overall, molecular classification identifies a prognostic gradient in UCS, with POLEmut tumors showing the most favorable outcomes and p53abn and NSMP tumors the least favorable. Nevertheless, apart from the POLEmut subgroup, UCS generally has poorer outcomes than other endometrial carcinoma histotypes within the same molecular category. Prognostic assessment should therefore incorporate both molecular classification and the histological characteristics of carcinosarcoma [44,45,46].

The successful implementation of ProMisE in diagnostic specimens allows for earlier informed decision-making, potentially guiding fertility-sparing strategies for young women or tailoring surgical staging and adjuvant therapy for high-risk patients before definitive surgery [9,10,18]. This molecular framework is now integral to international guidelines and the FIGO 2023 staging system, marking precision medicine in endometrial cancer [30,39].

### 5.3. Integration into Clinical Staging and Guidelines

Stage remains the strongest single determinant of outcome in EC. According to the most recent US cancer statistics, the 5-year relative survival rate for uterine corpus cancer is 81% overall, 95% for localized disease, 70% for regional disease, and 19% for distant disease [1]. These summary stages do not correspond exactly to FIGO stages: localized disease is confined to the uterus, regional disease has spread to adjacent organs or regional lymph nodes, and distant disease has metastasized to distant sites. Such anatomically based categories, however, do not capture the prognostic heterogeneity within each stage.

To address this limitation, the 2023 FIGO update incorporates histotype, LVSI, and molecular classification into staging, so that tumors confined to the uterus may be downstaged or upstaged based on molecular features, regardless of conventional histology or LVSI [16,39]. Stage I–II tumors harboring pathogenic POLE mutations, whether confined to the uterine corpus or with cervical extension, are classified as stage IAmPOLEmut, independent of histologic type, grade, or LVSI. Given their excellent prognosis, this often results in downstaging and may support de-escalation of adjuvant therapy [39,47]. In contrast, p53abn tumors with any degree of myometrial invasion are classified as stage IICmp53abn, regardless of invasion depth, cervical involvement, or LVSI; even low-grade tumors are upstaged because of the high risk of recurrence associated with this subtype [39,47]. The prognostic value of the revised system has been supported in validation cohorts [47].

Within each stage category, histotype further modifies prognosis. In a US statewide population-based registry study of more than 27,000 women, the age-standardized 5-year cause-specific survival was 85.3% for type I (low-grade endometrioid) carcinoma, but it was markedly lower for high-grade histotypes: 58.3% for high-grade endometrioid carcinoma, 55.1% for clear cell carcinoma, 38.0% for serous carcinoma, and 30.1% for carcinosarcoma [48].

Molecular classification adds prognostic information that is independent of stage and histotype, and it identifies patients whose outcome is compromised by under- or over-treatment. In the molecular analysis of PORTEC-3, all patients had high-risk stage I–III disease and received adjuvant radiotherapy with or without chemotherapy. Five-year recurrence-free survival (RFS) was 98% for POLEmut, 72% for MMRd, 74% for NSMP, and 48% for p53abn tumors, and 5-year OS was 98%, 81%, 89%, and 54%, respectively [42]. Notably, p53abn tumors were the only subgroup with a significant benefit from the addition of chemotherapy (5-year RFS 59% vs. 36%) [42]. Because p53abn disease also occurs in endometrioid and apparently early-stage tumors, histology-based risk assignment may under-treat these patients. Conversely, POLEmut tumors have an excellent prognosis even when high-grade [41], so histology-based escalation would over-treat them. These data provide the rationale for the molecular upstaging and downstaging introduced in FIGO 2023, and a similar molecular prognostic gradient is observed in carcinosarcoma (Section 5.2). By integrating tumor biology into surgical staging, the 2023 FIGO system represents a major shift toward precision medicine in EC: molecular diagnostics do not change tumor biology, but they improve prediction and prognosis within conventional subgroups by matching treatment intensity to biological risk.

## 6. Cellular and Molecular Mechanisms

### 6.1. Hormonal Signaling and Estrogen-Driven Carcinogenesis

EC, particularly the type I endometrioid subtype, is a typical hormone-dependent malignancy that arises in the setting of prolonged and unopposed estrogenic stimulation of the endometrial lining [18]. Chronic exposure to estradiol (E2) derived from endogenous or exogenous sources, results in sustained mitogenic signaling and promotes a stepwise progression from normal endometrium to hyperplasia, EIN, and ultimately adenocarcinoma [49]. In postmenopausal women, although ovarian estrogen production declines, adipose tissue becomes the predominant source of estrogen via aromatization of circulating androgens, thereby maintaining a hormonally permissive environment for tumorigenesis [49,50].

At the molecular level, estrogen exerts its oncogenic effects through both genomic and non-genomic signaling pathways [51]. In the classical genomic pathway, E2 binds to nuclear estrogen receptor alpha (ERα). The activated ERα then interacts with estrogen response elements (EREs) within promoter regions of target genes, recruiting co-regulatory complexes that drive the transcription of genes involved in cell cycle progression, including Cyclin D1, as well as oncogenes such as PIWIL1 [50,51,52]. In parallel, non-genomic signaling is mediated through membrane-associated receptors, including G protein-coupled estrogen receptors (GPR30/GPER-1), which initiate rapid intracellular signaling cascades such as the PI3K/AKT and MAPK/ERK pathways [52]. These pathways promote cellular proliferation, survival, and invasion independent of direct transcriptional regulation [51]. Notably, overexpression of GPER-1 has been associated with high-grade tumors and deep myometrial invasion, underscoring its role in aggressive disease phenotypes [52].

Emerging evidence highlights a critical synergistic crosstalk between estrogen and insulin/insulin-like growth factor-1 (IGF-1) signaling in the pathogenesis of EC [21,51]. Both estrogen excess and hyperinsulinemia act as independent risk factors, and their coexistence—commonly observed in obesity and metabolic syndrome—amplifies oncogenic signaling [53]. Hyperinsulinemia reduces circulating levels of sex hormone-binding globulin (SHBG) and insulin-like growth factor binding proteins (IGFBPs), thereby increasing the bioavailability of free E2 and IGF-1 [21]. At the receptor level, estrogen can induce phosphorylation of insulin receptor-β (InsR-β) and insulin receptor substrate-1 (IRS-1), while insulin signaling can activate ERα in a ligand-independent manner [51]. This synergy hyperactivates downstream signaling pathways, including AKT, MAPK, and ERK, resulting in increased S-phase cell distribution and suppression of apoptosis [50,51].

In addition to epithelial-intrinsic mechanisms, stromal–epithelial interactions play a critical role in regulating endometrial homeostasis and carcinogenesisr [54]. Estrogen-driven epithelial proliferation is largely mediated indirectly through stromal ERα signaling, which stimulates the production of paracrine growth factors such as IGF-1 and fibroblast growth factors (FGFs) [18,54]. Progesterone acts as a critical physiological antagonist by binding to stromal progesterone receptors (PR), inducing the transcription factor Hand2, which suppresses FGF production and thereby inhibits epithelial proliferation [54]. Furthermore, progesterone signaling promotes the synthesis of retinoic acid (RA) within stromal cells. RA acts in a paracrine manner on epithelial cells to induce the expression of 17β-hydroxysteroid dehydrogenase type 2 (HSD17B2), an enzyme that converts biologically active E2 into the less potent estrone, effectively attenuating local estrogenic stimulation [55]. Disruption of these protective stromal mechanisms—through progesterone resistance, loss of PR expression, or stromal depletion during tumor progression—removes critical regulatory constraints and facilitates malignant transformation [54,56].

Collectively, these findings underscore that endometrial carcinogenesis is not solely driven by estrogen excess but rather by a complex interplay between hormonal signaling, metabolic dysregulation, and microenvironmental interactions. Understanding these integrated pathways provides a critical framework for the development of targeted therapies and preventive strategies in hormone-driven endometrial cancer.

### 6.2. Key Signaling Pathways

**Figure 2:**
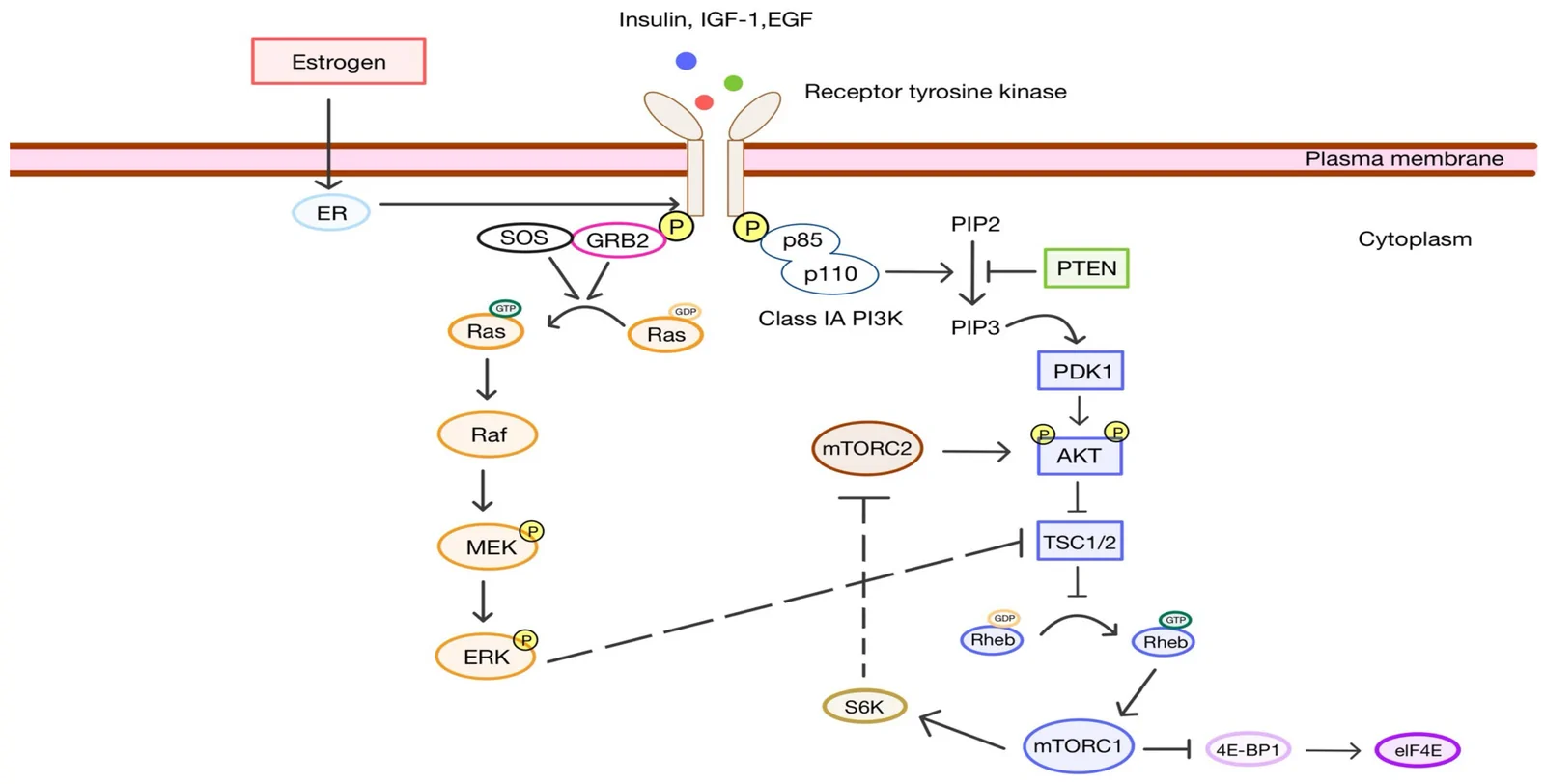
**Overview of the PI3K/AKT/mTOR and Raf/MEK/ERK signaling pathways. Arrows: promote or activate; T lines (solid and dotted lines): inhibit or block.** IGF-1, insulin-like growth factor1; EGF, epidermal growth factor; PI3K, phosphatidylinositol3 kinase; PTEN, phosphatase and tensin homolog, PDK, phosphotidylinositide-dependent kinases 1; AKT, v-akt murine thymoma viral oncogene homolog 1; TSC, tuberous sclerosis protein; Rheb, RAS homolog enriched in the brain; mTORC, mechanistic target of rapamycin complex, 4EBP1, eukaryotic initiation factor 4E-binding protein 1; elF4E, eukaryotic translation initiation factor 4E; S6K, ribosomal S6 kinase; ER, estrogen receptor.

An overview of the PI3K/AKT/mTOR and Raf/MEK/ERK signaling pathways is illustrated in Figure 2.

#### 6.2.1. PI3K/AKT/mTOR Pathway

The PI3K/AKT/mTOR signaling pathway represents the most frequently dysregulated molecular network in EC, with alteration rates ranging from 80% to 92% in endometrioid types [57]. This pathway serves as a central regulatory hub for metabolic signaling, sensing nutrient levels and growth factors to regulate protein synthesis, cell cycle progression, and survival [58].

Activation of the pathway is initiated by the binding of extracellular ligands—such as insulin, IGF-1, and epidermal growth factor (EGF)—to receptor tyrosine kinases (RTKs) or G protein–coupled receptors (GPCRs) located on the cell membrane [59,60]. This interaction facilitates the recruitment and activation of class IA phosphatidylinositol 3-kinase (PI3K), a heterodimer composed of a p85 regulatory subunit and a p110 catalytic subunit. Upon activation, the p110 subunit catalyzes the phosphorylation of phosphatidylinositol-4,5-bisphosphate (PIP2) to generate phosphatidylinositol-3,4,5-trisphosphate (PIP3), a critical lipid second messenger [60,61].

PIP3 serves as a docking platform for proteins containing pleckstrin homology (PH) domains, most notably AKT and phosphoinositide-dependent kinase-1 (PDK1) [59]. Full activation of AKT requires dual phosphorylation: PDK1 phosphorylates AKT at threonine 308 (Thr308), while the mTOR complex 2 (mTORC2) phosphorylates serine 473 (Ser473) [61]. Once activated, AKT phosphorylates and inhibits the tuberous sclerosis complex (TSC1/TSC2), a key negative regulator of the small GTPase Rheb. Inactivation of TSC2 allows Rheb to accumulate in its active GTP-bound state, which in turn activates mTOR complex 1 (mTORC1) at the lysosomal surface [57,59,61].

Activated mTORC1 promotes cellular growth and protein synthesis through phosphorylation of downstream effectors, including p70S6 kinase (S6K1) and eukaryotic translation initiation factor 4E-binding protein 1 (4E-BP1) [61]. Phosphorylation of 4E-BP1 releases its inhibitory interaction with eIF4E, thereby facilitating cap-dependent mRNA translation and promoting tumor cell proliferation [58].

The activity of the PI3K/AKT/mTOR pathway is tightly regulated by the tumor suppressor PTEN, which acts as the essential “gatekeeper” by dephosphorylating PIP3 back into PIP2, thereby effectively shutting down AKT recruitment and the entire downstream cascade [59,60,62]. In endometrial cancer, loss of PTEN function is one of the most common molecular alterations and represents a key driver of constitutive pathway activation [62,63].

Furthermore, this signaling network is modulated by complex negative feedback loops. For example, activated S6K1 can phosphorylate and inhibit IRS-1, which normally serves to dampen upstream signaling from the insulin receptor [3,61]. However, in malignant cells, these feedback mechanisms are frequently disrupted, contributing to sustained pathway activation, uncontrolled proliferation, and resistance to targeted therapies [58].

Pathologically, hyperactivation of the PI3K/AKT/mTOR axis confers several oncogenic advantages. AKT promotes cell survival by inhibiting pro-apoptotic factors such as BAD and caspase-9, while suppressing transcription factors of the FOXO family that regulate apoptosis-related gene expression [3,64]. In addition, the pathway drives metabolic reprogramming toward aerobic glycolysis (the Warburg effect), enhancing glucose uptake and biosynthetic capacity [59,65]. Clinically, persistent activation of this pathway has been implicated in progestin resistance in EC, partly through inhibition of autophagy. Notably, this resistance may be partially reversed by mTOR inhibitors such as everolimus, highlighting the therapeutic relevance of targeting this pathway [66].

#### 6.2.2. p53 Pathway

The p53 signaling pathway serves as a master regulator of genomic integrity, orchestrating cellular responses to stress, such as DNA damage, hypoxia, and oncogenic signaling [40]. In EC, abnormalities of this pathway are particularly characteristic of the p53abn molecular subtype, which corresponds to the CNH genomic category identified by TCGA. This subtype is characterized by a high prevalence of TP53 mutations, occurring in more than 90% type II EC, and represents the most aggressive clinical phenotype with the poorest overall outcome [8,11,21].

Under normal physiological conditions, p53 acts as a transcription factor that preserves genomic stability by regulating both the G1/S and G2/M cell cycle checkpoints [38,67]. In response to DNA damage, wild type p53 accumulates and activates the transcription of key target genes, including CDKN1A (p21) for cell cycle arrest, GADD45 for DNA repair, and pro-apoptotic factors such as BAX and BAK [66,68].

Activation of p21 inhibits cyclin-dependent kinases (CDKs), halting the transition from the G1 to the S phase [68]. Simultaneously, p53 signaling modulates the cdc2/cdc25C axis to govern the G2/M transition. The G2/M checkpoint is sustained when inhibitory kinases phosphorylate cell division control 2 (cdc2) at the Tyr15 site. Conversely, mitotic entry is triggered when the cdc25C phosphatase removes this inhibitory phosphate, thereby activating cdc2 [67].

However, loss of functional p53 through mutation or deletion results in profound genomic instability and uncontrolled cellular proliferation [8,67]. A critical consequence of p53 deficiency is the loss of the G1/S checkpoint, rendering these tumor cells uniquely dependent on the G2/M checkpoint to survive in the presence of accumulated DNA damage [38]. This dependency creates a therapeutic vulnerability that has attracted increasing clinical interest. Pharmacologic inhibition of G2/M checkpoint regulators, including Wee1 kinase inhibitors such as adavosertib and inhibitors targeting Chk1/Chk2, can force p53-deficient tumor cells to skip arrest and enter mitosis prematurely, leading to mitotic catastrophe and apoptosis [18,38].

In addition to checkpoint dysregulation, EC cells with loss-of-function p53 mutations often activate compensatory survival pathways. One such pathway is the P38/MK2 signaling cascade, which helps maintain G2/M checkpoint function and confers resistance to chemotherapy-induced stress [22,67]. Furthermore, beyond the loss of its original tumor suppressor function, certain missense mutations confer “gain-of-function” (GOF) properties, further promoting epithelial–mesenchymal transition (EMT), increased tumor invasiveness, and resistance to radiotherapy and chemotherapy [8,67,69].

The activity and stability of the p53 pathway are further fine-tuned by various intracellular factors. At the protein level, the oncogenic protein Gankyrin (PSMD10) suppresses p53 signaling by increasing the ubiquitination and subsequent proteasomal degradation of p53 through its interaction with the MDM2 E3 ligase [52]. In contrast, the tumor suppressor ENKUR has been reported to stabilize p53 expression to inhibit tumor metastasis [70].

Overall, p53 deficiency in aggressive endometrial cancer triggers a critical dependency on the G2/M checkpoint. This molecular vulnerability, alongside compensatory P38/MK2 signaling, facilitates targeted therapies like Wee1 inhibitors and overcome chemoresistance in p53-mutant malignancies.

#### 6.2.3. Wnt/β-Catenin Pathway

**Figure 3:**
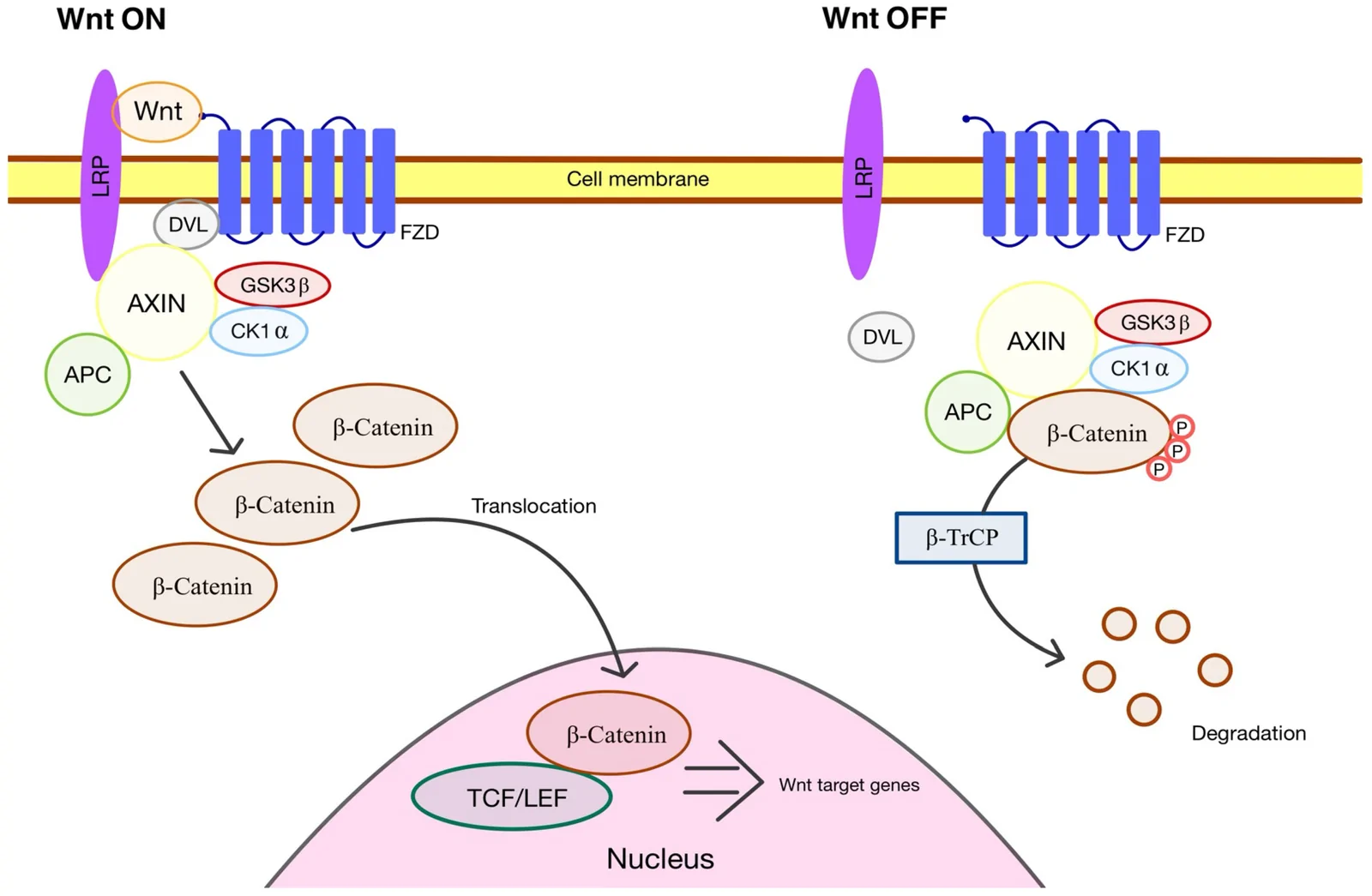
**Regulation of the Wnt/β-catenin signaling pathway. Arrows: promote or activate.** Wnt signaling exists in “off” and “on” states. In the absence of Wnt ligands, β-catenin is phosphorylated by the AXIN-centered destruction complex and subsequently degraded via β-TrCP-mediated ubiquitination. Upon Wnt binding to the FZD-LRP5/6 receptor complex, the destruction complex is inactivated, resulting in β-catenin accumulation and nuclear translocation, where it activates TCF/LEF-mediated transcription of Wnt target genes. LRP, low-density lipoprotein receptor-related protein; FZD, Frizzled; DVL, Dishevelled; GSK3β, APC, Adenomatous Polyposis Coli; glycogen synthase kinase 3β; CK1α, casein kinase 1α; TCF, T-cell factor; LEF, lymphoid enhancer factor; β-TrCP, β-transduction repeat-containing E3 ubiquitin-protein ligase.

The Wnt/β-catenin signaling pathway, also known as the canonical Wnt pathway, is a fundamental regulator of cellular proliferation, differentiation, and tissue homeostasis that is aberrantly activated in approximately 65% of EC cases [71]. This pathway is particularly significant in the CNL molecular subtype, where it acts as a primary oncogenic driver [3]. Figure 3 depicted the detailed mechanisms of Wnt/β-catenin pathway responsible for tumor genesis and disease progression.

The signaling cascade is initiated when extracellular Wnt ligands, such as WNT7A or WNT2, bind to a receptor complex composed of a Frizzled (FZD) transmembrane receptor and the co-receptors low-density lipoprotein receptor-related protein 5/6 (LRP5/6) [71]. This binding induces the recruitment and sequestration of Dishevelled (DVL) proteins to the plasma membrane. Activation of DVL leads to inhibition and disassembly of the β-catenin destruction complex, which is normally composed of Axin, Adenomatous Polyposis Coli (APC), Glycogen Synthase Kinase 3β (GSK3β), and Casein Kinase 1α (CK1α) [72]. In the absence of an active destruction complex, free β-catenin (the protein product of CTNNB1) accumulates in the cytoplasm and translocates into the nucleus. This translocation is often facilitated by co-factors such as BCL9 [71,73,74]. In the nucleus, β-catenin interacts with T-cell factor (TCF) and lymphoid enhancer factor (LEF) transcription factors, displacing corepressors to activate the expression of oncogenic target genes, including Cyclin D1 (CCND1), c-Myc, CD44, and AXIN2 [68,71,75].

In endometrial carcinogenesis, activation of the Wnt pathway frequently occurs through intrinsic genetic alterations that bypass the requirement for extracellular Wnt stimulation. Among these, hotspot mutations in Exon 3 of CTNNB1, particularly involving residues such as Ser33 and Ser37, are observed in approximately 20–25% of endometrioid ECs [71,72,73]. These mutations prevent the phosphorylation and subsequent ubiquitination of β-catenin by the destruction complex, leading to constitutive pathway activation [73].

In addition to CTNNB1 mutations, multiple molecular alterations further enhance β-catenin stability and signaling activity. Activating KRAS mutations can stabilize β-catenin through inhibition of GSK3β activity [15]. Loss of the transcription factor SOX17, which normally promotes proteasomal degradation of β-catenin, is another recurrent event in the CNL subgroup that contributes to pathway hyperactivation [15]. Furthermore, the RNA-binding protein MEX3A has been shown to interact with DVL3, thereby stabilizing β-catenin and enhancing downstream transcriptional activity [76]. Recent studies have also identified MRP4 (ABCC4) as a novel regulator capable of directly interacting with β-catenin to prevent its degradation independently of its conventional drug transporter function [77]. Conversely, the tumor suppressor ENKUR facilitates recruitment of the E3 ubiquitin ligase FBXW7 to promote β-catenin ubiquitination and degradation; therefore, loss of ENKUR contributes to β-catenin accumulation and tumor progression [70].

The Wnt/β-catenin pathway is also closely integrated with hormonal regulation in the endometrium. During the proliferative phase of the menstrual cycle, estrogen stimulates Wnt signaling to promote endometrial glandular proliferation and tissue expansion [71,78]. In contrast, progesterone antagonizes Wnt activity through induction of Dickkopf-1 (DKK1), a secreted Wnt inhibitor, and the transcription factor FOXO1, both of which facilitate β-catenin degradation and cellular differentiation [73,75]. Loss of progesterone-mediated suppression is considered a crucial event in endometrial tumorigenesis [71,73]. Together, these findings underscore the pivotal role of the Wnt/β-catenin pathway in the molecular pathogenesis and progression of endometrial cancer.

#### 6.2.4. MAPK Pathway (Figure 2)

The Mitogen-Activated Protein Kinase (MAPK) pathway, specifically the classical Ras/Raf/MEK/ERK cascade, is a vital oncogenic signaling network that transmits extracellular signals from the cell surface to the nucleus to regulate gene expression, cell cycle progression, and survival [59,68]. In EC, this pathway is frequently hyperactivated, primarily through KRAS mutations, which are found in approximately 15% to 30% of Type I (endometrioid) cases [15,28].

The signaling cascade is typically initiated by the binding of growth factors, such as EGF and IGF-1, to receptor tyrosine kinases (RTKs) located on the cell surface [60,64]. Upon receptor activation, adaptor proteins like Grb2 and guanine nucleotide exchange factors (GEFs) such as Sos are recruited to the plasma membrane. These factors facilitate the loading of Ras with GTP, shifting it from an inactive to an active state [64,68].

Activated Ras subsequently recruits Raf (specifically B-Raf or C-Raf) to the membrane for activation. Raf then phosphorylates and activates MEK1/2, a dual-specificity kinase that in turn phosphorylates extracellular signal-regulated kinase 1/2 (ERK1/2) [59]. Phosphorylated ERK (p-ERK) translocates into the nucleus where it activates numerous transcription factors, including Elk-1, c-Fos, c-Jun, and c-Myc [68,79]. These factors drive the expression of genes essential for the G1/S transition, such as Cyclin D1, thereby promoting unchecked cellular proliferation [60,68].

The MAPK pathway serves as a central integration point for hormonal stimuli in the endometrial microenvironment. Estrogen acts through estrogen receptor alpha (ERα) to rapidly activate the MAPK/ERK signaling branch, often via non-genomic membrane-initiated signals or synergistic crosstalk [51]. Additionally, chronic hyperinsulinemia—a hallmark of obesity-related EC—synergistically promotes progression by activating the MAPK/ERK pathway through insulin receptor-beta (InsRβ) and insulin receptor substrate-1 (IRS-1). This crosstalk enhances the mitogenic effects of estrogen, leading to increased S-phase cell distribution and survival [51].

Beyond its role in proliferation, specific branches and modulators of the MAPK pathway drive aggressive phenotypes. Autocrine Motility Factor (AMF), which is highly expressed in EC, activates the MAPK/ERK pathway to promote the epithelial–mesenchymal transition (EMT). This process involves the upregulation of Snail and TGFBR1 and the loss of E-cadherin, facilitating tumor invasion and metastasis [69].

In addition to the classical ERK pathway, stress-activated MAPK branches including JNK and p38 are also implicated in endometrial carcinogenesis. For instance, Lanosterol Synthase (LSS), a key enzyme in cholesterol metabolism, has been reported to promote EC cell proliferation and inhibit apoptosis through activation of the MAPK/JNK signaling axis [80]. These findings highlight the diverse biological functions of MAPK signaling beyond cell proliferation alone.

Importantly, the MAPK pathway demonstrates extensive crosstalk with the PI3K/AKT/mTOR pathway, another major oncogenic signaling network in EC [40,59]. Inhibition of the PI3K pathway may lead to compensatory activation of MAPK signaling, thereby maintaining tumor cell survival and contributing to therapeutic resistance. This bidirectional interaction has significant clinical implications and suggests that combined blockade of both MAPK and PI3K/AKT/mTOR pathways may represent a more effective therapeutic strategy in selected patients with endometrial cancer [59,60].

### 6.3. Tumor Microenvironment (TME)

The endometrial tumor microenvironment (TME) is a complex and dynamic ecosystem comprising malignant cells, stromal cells, immune cells, endothelial cells, and the extracellular matrix (ECM) [81]. This TME actively enhances tumor cell proliferation and invasion, transitioning from a state of immune surveillance in normal tissue to immune evasion during cancer progression [82]. A hallmark of the EC TME is metabolic reprogramming, where tumor cells exhibit increased aerobic glycolysis (the Warburg effect), leading to a lactate-rich environment [65]. This extracellular lactate triggers the M2 polarization of tumor-associated macrophages (TAMs), which in turn secrete cytokines like IL-6 to promote the epithelial–mesenchymal transition (EMT), angiogenesis, and metastasis [65].

The tumor immune microenvironment (TIME) exhibits significant heterogeneity correlated with molecular subtypes [83]. POLE-mutated and MSI-H subtypes are characterized as “immune-included” or “inflamed” tumors, displaying high TMB, abundant neoantigens, and dense CD8+ cytotoxic T-cell infiltration [82]. In contrast, the copy-number high (CNH/p53abn) subtype are often “immune-suppressed”, enriched with regulatory T cells (Tregs) and M2-like macrophages that facilitate immune escape [82,83]. Meanwhile, copy-number low (CNL/NSMP) subtypes typically represent “immune-desert” or “excluded” phenotypes with restricted immune cell infiltration [82,84].

### 6.4. Epigenetic Regulation

Endometrial cancer is increasingly recognized as an epigenetic disease where gene function is impaired through non-mutational mechanisms that alter chromatin accessibility [85]. One of the most significant recent discoveries is the transcriptional silencing of PAX2, a potent tumor suppressor inactivated in over 80% of ECs [86]. This silencing occurs via a distinct “pearl necklace” model of epigenetic reprogramming, where open/active chromatin features (H3K27ac/H3K4me3) at a superenhancer are replaced by inaccessible/repressive chromatin (H3K27me3) within cohesin-mediated 3D genomic loops. This focal epigenetic event is considered a very early, initiating driver of endometrial carcinogenesis [86].

Importantly, unlike irreversible genetic mutations, epigenetic alterations are reversible. This reversibility has generated potential for the development of targeted therapies utilizing DNA methyltransferase (DNMT) inhibitors, HDAC inhibitors, and other epigenetic modifiers. Such strategies may provide novel therapeutic opportunities for personalized treatment in endometrial cancer [85,87].

## 7. Genomic and Emerging Technologies

The diagnostic landscape of EC has undergone a profound transformation from conventional histomorphology assessment to integrated molecular characterization [11]. This paradigm shift was largely initiated by TCGA, which established the molecular classification system of EC based on somatic mutational burden and SCNAs [6,88]. Advances in genomic technologies have since enabled increasingly precise tumor profiling, improving diagnostic accuracy, prognostic stratification, and therapeutic decision-making. In parallel, emerging technologies are being developed to provide minimally invasive, objective, and real-time diagnostic alternatives that may further advance precision medicine in EC.

### 7.1. Genomic Techniques

Among current molecular diagnostic approaches, Next-Generation Sequencing (NGS) has become one of the most transformative technologies in EC research and clinical practice. NGS provides high-throughput and highly sensitive detection of single-nucleotide variants (SNVs), insertions/deletions, and structural alterations within tumor genomes [3]. It is currently the standard method for identifying pathogenic POLE mutations in the exonuclease domain, which define the ultramutated molecular subgroup associated with excellent prognosis [9,89]. Comprehensive targeted sequencing panels, such as MSK-IMPACT, enable simultaneous analysis of up to 505 cancer-related genes, including PTEN, PIK3CA, and TP53, thereby facilitating integrated molecular classification and therapeutic target identification [90].

Because the cost and technical complexity of whole-exome and whole genome sequencing limit their routine implementation in clinical practice, more pragmatic surrogate approaches have been developed [10,16]. One widely adopted system is the ProMisE, which combines IHC for MMR proteins and p53 with targeted sequencing of pathogenic POLE hotspot mutations, commonly involving exons 9, 13, and 14. This hybrid approach provides a clinically feasible and cost-effective alternative for molecular classification while maintaining strong prognostic accuracy [9]. To further improve the accessibility and turnaround time of molecular testing, new technologies like QPOLE (multiplex genotyping PCR) and droplet digital PCR (ddPCR) are being developed to provide even faster (4–6 h) and cheaper alternatives for POLE testing [7,42].

### 7.2. Emerging Technologies

Beyond conventional genomic profiling, several emerging technologies are being investigated to improve the early detection, prognostic assessment, and longitudinal monitoring of endometrial cancer through minimally invasive and highly sensitive approaches.

One of the most promising developments is the use of liquid biopsy and fragmentomics. These techniques analyze cell-free DNA (cfDNA) and circulating tumor DNA (ctDNA) fragmentation patterns, including fragment size, distribution, and nucleotide motifs, using low-pass WGS combined with machine learning algorithms [91]. This non-invasive blood-based approach allows for early detection, with reported area under the curve (AUC) values reaching 0.96, while also enabling longitudinal monitoring of treatment response, and detection of minimal residual disease [5,91]. However, several limitations remain, including the extremely low abundance of tumor-derived DNA in early-stage EC and the lack of standardized testing protocols across institutions [91,92]. Nevertheless, future applications of longitudinal ctDNA kinetic analysis may potentially replace invasive tissue biopsies for monitoring tumor recurrence and identifying therapeutic resistance before the emergence of clinical or radiologic evidence [93].

Collectively, these genomic and emerging technologies are driving a transition toward precision oncology in endometrial cancer. Continued integration of molecular profiling, multi-omics platforms, artificial intelligence, and real-time diagnostic tools is expected to improve early detection, refine prognostic stratification, and facilitate individualized therapeutic strategies in the future management of EC.

## 8. Clinical Management

The management of EC has entered the era of precision medicine, integrating molecular and genomic characteristics into clinical decision-making [16]. While surgical resection remains the primary curative modality for localized disease, recent advancements in tumor profiling have redefined the selection of adjuvant treatments to optimize locoregional control and minimize toxicity [7,16,21]. For patients presenting with advanced or recurrent disease, the therapeutic landscape has been transformed by the emergence of novel systemic agents [28,94]. This section provides an overview of current clinical management strategies, from established surgical and radiation practices to the paradigm-shifting roles of targeted therapies and immunotherapy supported by the latest clinical trial data.

### 8.1. Surgical Treatment

Surgical removal of the primary tumor with a total hysterectomy and bilateral salpingo-oophorectomy (TH-BSO) remains the cornerstone of management for EC [21,40]. For early-stage disease confined to the uterus, minimally invasive surgery (MIS), including both traditional laparoscopy and robotic-assisted laparoscopy, is the preferred approach as it offers equivalent oncological outcomes to laparotomy with reduced perioperative morbidity and shorter hospital stays [5,16,18,21]. Sentinel lymph node (SLN) mapping with ultrastaging has increasingly replaced systematic pelvic and para-aortic lymphadenectomy for staging in low- and intermediate-risk disease, providing accurate nodal status while minimizing the risk of lower-extremity lymphedema [5,16]. In advanced stages (FIGO III-IV), primary cytoreductive surgery aiming for complete resection of all macroscopic disease is recommended whenever feasible [6,57,61]. For young patients with low-grade, early-stage endometrioid EC desiring future childbearing, fertility-sparing management using high-dose progestins or levonorgestrel-releasing intrauterine devices (LNG-IUD) may be offered after extensive counseling [5,18,95].

Molecular classification is also relevant to nodal staging, because the risk of occult lymph node metastasis differs among molecular subgroups. In a single-institution cohort of 172 patients who underwent primary surgery with planned pelvic with or without para-aortic lymphadenectomy and were retrospectively classified using ProMisE, nodal metastases were present in 44.8% of p53abn tumors, compared with 14.2% of POLEmut, 14.9% of MMRd, and 10.8% of NSMP tumors; on multivariate analysis, molecular subtype, but not preoperative grade or histotype, remained associated with nodal metastasis [96]. Among 756 patients with apparently uterine-confined EC staged by SLN mapping, copy-number-high/p53abn tumors carried an approximately three-fold higher risk of SLN micro- or macrometastases (odds ratio 3.1), alongside LVSI and deep myometrial invasion; however, no molecular subgroup had a rate of SLN metastasis low enough to justify omission of nodal assessment [97].

Occult nodal disease is not confined to p53abn tumors: in a multicenter cohort of 237 patients with clinical stage I EC and negative preoperative CT or PET-CT, occult nodal metastases were found in 19% of MMR-deficient (MSI) tumors versus 6.7% of MMR-proficient tumors, and this association persisted after adjustment for grade and LVSI and was most pronounced with combined or MLH1/PMS2 loss [98]. Taken together, these predominantly retrospective data indicate that molecular classification, which can be performed on preoperative endometrial biopsies, may help to anticipate the risk of occult nodal disease and to plan surgical staging, particularly in p53abn and MMRd tumors. At present, however, they support SLN assessment across all molecular subgroups rather than molecularly guided omission of nodal staging, which would require prospective validation.

### 8.2. Adjuvant Therapy

The selection of adjuvant treatment is guided by risk stratification based on clinicopathological factors, including age, stage, grade, lymphovascular space invasion (LVSI), and myometrial invasion, and increasingly incorporates molecular classification [6,99]. For patients with low-risk EC, adjuvant therapy is generally not indicated, as 5-year survival rates reach 95% with surgery alone [18]. In intermediate- and high-intermediate-risk EC, vaginal brachytherapy (VBT) or external beam radiation therapy (EBRT) are used to optimize locoregional control [16,40]. The phase III PORTEC-4a trial report, published in 2026, evaluated molecular-profile-directed adjuvant therapy in patients with high-intermediate-risk endometrial cancer. The findings support the feasibility of incorporating molecular profiling into risk stratification and adjuvant treatment decision-making [37].

For patients with high-risk EC at stage I–III, the randomized phase III PORTEC-3 trial demonstrated that adjuvant concurrent chemoradiotherapy (CCRT) followed by adjuvant chemotherapy (carboplatin/paclitaxel) significantly improves 10-year overall survival (74.4% vs. 67.3%) and recurrence-free survival compared to EBRT alone. Notably, the greatest absolute benefit from the addition of chemotherapy was observed in patients with p53-abnormal (p53abn) tumors in a post hoc molecular analysis [99,100,101]. Conversely, the randomized phase III GOG-258 trial, conducted in the adjuvant setting in patients with stage III–IVA disease not selected by molecular subgroup, found that although CCRT improved locoregional control compared with chemotherapy alone, it did not significantly prolong overall survival in advanced disease because of persistent distant recurrences [18,102].

### 8.3. Hormonal Therapy

Hormonal therapy (HT) is an attractive front-line option for patients with recurrent, low-grade, hormone receptor-positive (ER+/PR+) endometrioid EC, particularly those with low-volume or indolent disease [16,57]. Progestins (megestrol acetate or medroxyprogesterone acetate) are the most used agents, with objective response rates (ORR) ranging from 15% to 37% [40,57]. Other options include aromatase inhibitors (AIs) like letrozole, selective estrogen receptor modulators (SERMs) such as tamoxifen, and selective estrogen receptor degraders (SERDs) like fulvestrant [28,100]. While HT is well-tolerated, its efficacy as a single agent is often limited by the development of resistance, leading to investigations of combination strategies [21,103].

### 8.4. Targeted Therapy

Targeted therapies are increasingly integrated into the management of EC, designed to interfere with specific molecular pathways essential for tumor growth and progression [61]. The selection of these agents is increasingly guided by the tumor’s genomic profile, moving away from a uniform histopathological approach toward personalized medicine [3].

Among currently available targeted approaches, PI3K/AKT/mTOR pathway inhibitors have received considerable attention because this pathway represents the most frequently altered signaling cascade in EC, occurring in up to 80–95% of endometrioid cases [16,28]. Although monotherapy with mTOR inhibitors such as temsirolimus, everolimus, and ridaforolimus showed modest activity, with objective response rates (ORR) ranging from 4% to 25%, greater efficacy has been observed when these agents are combined with hormonal therapy [57,104]. In the noncomparative randomized phase II GOG-3007 trial, which enrolled patients with advanced or recurrent EC who had received no more than one prior chemotherapy regimen, the combination of everolimus and letrozole achieved a median PFS of 28 months in chemotherapy-naïve patients, compared with 4 months in those previously treated with chemotherapy. Based on randomized phase II evidence, everolimus plus letrozole has been incorporated into the NCCN Guidelines as a preferred hormonal therapy option for recurrent endometrioid endometrial cancer [105,106]. Similarly, in the randomized phase I/II VICTORIA trial in hormone receptor-positive recurrent or metastatic EC, the combination of vistusertib (a dual mTORC1/2 inhibitor) and anastrozole resulted in a median PFS of 5.2 months and an ORR of 24.5%. However, several limitations of this study should be considered, including the relatively small sample size and the lack of data on the level of hormone receptor expression. Furthermore, this therapeutic approach remains investigational and has not yet been incorporated into the NCCN guidelines as a standard treatment option [106,107].

Another important target is HER2, as HER2 (ERBB2) overexpression or amplification is found in 25–30% of serous ECs and carcinosarcomas [16,40]. A landmark randomized phase II trial by Fader et al. demonstrated that adding trastuzumab to standard carboplatin/paclitaxel in patients with primary advanced (stage III–IV) or recurrent HER2-positive uterine serous carcinoma, mainly in the first-line setting, significantly improved median PFS from 8.0 to 12.6 months (HR 0.44; P = 0.005) [102]. More recently, antibody-drug conjugates (ADCs) have shown potent activity [16]. This combination is now a preferred NCCN regimen for HER2-positive stage III–IV or recurrent serous carcinoma or carcinosarcoma [106]. In the second-line or later setting, the single-arm phase II DESTINY trial of previously treated HER2-expressing (IHC 2+ or 3+) solid tumors showed that trastuzumab deruxtecan (T-DXd) achieved an overall response rate of 57.5% in HER2-expressing EC, with responses as high as 84.6% in the IHC 3+ subgroup [108]. Likewise, in the single-arm phase II STATICE trial of uterine carcinosarcoma previously treated with chemotherapy, T-DXd demonstrated an ORR of 54.5% in HER2-high and 70% in HER2-low uterine carcinosarcomas [109]. T-DXd is now an NCCN-recommended biomarker-directed therapy for previously treated HER2-positive (IHC 2+ or 3+) EC and for carcinosarcoma with any HER2 expression (IHC 1+ to 3+) [106] and has received accelerated FDA approval for HER2 IHC 3+ solid tumors. However, current evidence is largely based on single-arm phase II studies, and HER2 scoring in EC remains unstandardized. Further studies are needed to clarify treatment-related toxicities and establish EC-specific HER2 scoring criteria [16].

In addition, targeting cell cycle regulation with CDK4/6 inhibitors has emerged as a promising strategy for estrogen receptor-positive (ER+) EC [40]. The randomized phase II PALEO trial in ER-positive endometrioid EC that was primary metastatic or had relapsed after at least one prior systemic therapy (mostly second-line or later) showed that the combination of palbociclib and letrozole significantly improved PFS compared to letrozole alone (8.3 vs. 3.0 months, HR 0.56) [103]. Similarly, abemaciclib plus letrozole achieved an ORR of 30% and a median PFS of 9.1 months in a single-arm phase II study of heavily pretreated ER-positive recurrent EC (median of three prior lines), with all responses occurring in endometrioid tumors and responses observed irrespective of MMR status. Notably, ctDNA analysis from these trials suggests that PI3K pathway activation may be a common mechanism of acquired resistance to CDK4/6 inhibition [110]. Based on phase II evidence, CDK4/6 inhibitors combined with endocrine therapy are listed by the NCCN as “useful in certain circumstances” for ER-positive recurrent disease [106].

Another emerging therapeutic strategy involves nuclear export inhibitors. Selinexor is an oral inhibitor of exportin 1 (XPO1) that promotes the nuclear retention of tumor suppressor proteins like p53 [16,40]. In the randomized, placebo-controlled phase III SIENDO trial, selinexor was evaluated as maintenance therapy after platinum-based chemotherapy in patients with primary stage IV or recurrent EC [40,111]. While the intention-to-treat population showed modest benefit, a prespecified subgroup analysis of TP53 wild-type tumors showed a dramatic improvement in median PFS (28.4 vs. 5.2 months when compared with placebo; HR 0.41) [28,111]. This benefit was observed regardless of mismatch repair (MMR) status [28]. However, this subgroup analysis was not adjusted for multiplicity, and most patients had received chemotherapy alone as first-line treatment, limiting its applicability to current ICI-based treatment settings. Accordingly, selinexor maintenance for TP53 wild-type EC remains investigational and is not currently included in the NCCN recommendations.

Furthermore, PARP inhibitors and synthetic lethality have become an area of growing interest, particularly in p53-abnormal (p53abn) or PTEN-deficient tumors that may exhibit homologous recombination deficiency (HRD) [102,112]. The randomized, placebo-controlled phase IIb UTOLA trial reported that olaparib maintenance after platinum-based chemotherapy for advanced or metastatic disease improved mPFS in HRD-positive tumors compared to placebo (5.4 vs. 3.6 months, HR 0.59) [113]. In the first-line randomized phase III DUO-E trial in primary advanced or recurrent EC, the addition of olaparib maintenance to frontline durvalumab and chemotherapy further enhanced PFS, particularly in the pMMR/MSS group (15.1 vs. 9.6 months) [114]. Accordingly, durvalumab plus olaparib maintenance is listed in the NCCN Guidelines as a Category 2B option for pMMR tumors, whereas olaparib monotherapy is not currently recommended [106].

Beyond these approaches, anti-angiogenic agents and other novel targets continue to expand the therapeutic landscape of EC. Bevacizumab (anti-VEGF) has shown activity when combined with chemotherapy, a post hoc analysis of the first-line randomized phase II GOG-86P trial suggesting a greater benefit in patients with TP53 mutant tumors, although the trial was not powered for this comparison [115]. Other investigative targets include TROP2 (e.g., sacituzumab govitecan achieved a 22% ORR in the single-arm phase II TROPiCS-03 basket study in patients previously treated with platinum-based chemotherapy and anti-PD-(L)1 therapy) and folate receptor α (FRα) (e.g., mirvetuximab soravtansine) [116]. Furthermore, novel agents like adavosertib (a Wee1 inhibitor) have demonstrated deep responses in ~30% of recurrent serous EC [18,28]. These newer agents remain investigational. Sacituzumab govitecan is currently being evaluated in the phase III ASCENT-GYN-01 trial in patients previously treated with platinum-based chemotherapy and immunotherapy, whereas evidence for adavosertib is limited to single-arm phase II studies [116]. Neither agent is currently included in the NCCN recommendations for EC.

### 8.5. Immunotherapy

Immunotherapy, particularly immune checkpoint inhibitors (ICIs), has revolutionized the therapeutic landscape for advanced and recurrent EC. The rationale for its use is primarily driven by the high immunogenicity of specific molecular subtypes, such as MMRd or MSI-H tumors, which account for approximately 25–30% of all ECs [16,28,40]. These tumors exhibit a high TMB, resulting in increased neoantigen expression and robust infiltration by tumor-infiltrating lymphocytes (TILs), making them highly susceptible to anti-PD-1/PD-L1 blockade [94].

#### 8.5.1. Single-Agent Immune Checkpoint Blockade

The efficacy of monotherapy ICIs has been firmly established in the recurrent setting for MMRd/MSI-H patients. The single-arm phase II KEYNOTE-158 basket trial evaluated pembrolizumab as second-line or later therapy in previously treated MMRd/MSI-H advanced EC and reported an ORR of 48–57.1% and a median PFS of 25.7 months [117]. These findings led to the first tumor-agnostic FDA approval for pembrolizumab [83]. Similarly, the single-arm phase I GARNET trial demonstrated durable activity of dostarlimab in the second-line or later setting after platinum-based chemotherapy in MMRd tumors, with a confirmed ORR of 42.3–45.5% and a median DOR that was not reached, whereas the ORR in pMMR tumors was substantially lower (13.4–15.4%) [4,118]. Accordingly, pembrolizumab or dostarlimab monotherapy after platinum-based therapy is listed in the NCCN Guidelines as a biomarker-directed option [106]. However, in MMRd endometrial cancer, the KEYNOTE-158 and GARNET trials reported ORR of 48% and 42.3%, respectively, indicating that more than half of patients do not achieve an objective response to ICI monotherapy. A recent review highlighted that the mechanisms underlying primary resistance to ICIs in MMRd/MSI-H endometrial cancer remain poorly understood. Moreover, TMB and PD-L1 expression have shown inconsistent associations with treatment response, and no additional biomarkers, including TP53, BRCA, or homologous recombination deficiency (HRD), have been established as predictors of ICI response [119].

Additional studies have further supported the efficacy of single-agent ICIs in MMRd disease. Agents such as nivolumab achieved ORRs ranging from 23–46% in MMRd tumors, while durvalumab in the PHAEDRA trial demonstrated an ORR of 43% in MMRd tumors compared with only 3% in pMMR tumors. Likewise, avelumab showed an ORR of 26.7% in MMRd EC, reinforcing the importance of molecular selection for immunotherapy [16,18,83]. The NCCN Guidelines list avelumab, nivolumab, tislelizumab, and retifanlimab (category 2B) as additional options for MSI-H/MMRd disease based on smaller single-arm studies [106].

#### 8.5.2. Combination of Immunotherapy and Targeted/Anti-Angiogenic Agents

To address the relative resistance of pMMR or MSS tumors to single-agent ICIs, combination strategies have been developed. In the phase III KEYNOTE-775/Study 309 compared pembrolizumab plus lenvatinib with physician’s choice chemotherapy in patients who progressed following platinum-based therapy. The combination significantly improved median OS (18.3 vs. 11.4 months) and median PFS (7.2 vs. 3.8 months) across all-comers. In the pMMR population, in which this combination is particularly important, OS was 18.0 months versus 12.2 months, while PFS was 6.7 months versus 3.8 months [120]. This combination is a Category 1 NCCN option for previously treated pMMR disease [106]. However, as KEYNOTE-775 enrolled ICI-naive patients, and ICI plus chemotherapy is now a first-line standard, randomized evidence supporting its use after prior ICI therapy remains lacking.

#### 8.5.3. Frontline Chemo-Immunotherapy

Recent phase III trials have incorporated ICIs into the standard frontline regimen of carboplatin and paclitaxel as first-line treatment for primary advanced (stage III–IV) or recurrent EC, with dMMR and pMMR populations analyzed separately. In the RUBY trial (Part 1), the addition of dostarlimab to standard chemotherapy significantly reduced the risk of disease progression or death by 72% in MMRd patients (HR 0.28), resulting in a 24-month PFS rate of 61.4% compared with 15.7%. A benefit was also observed in the pMMR population (HR 0.76) [94,121,122].

Similarly, in the same first-line setting, the randomized phase III NRG-GY018 trial demonstrated that pembrolizumab plus chemotherapy provided a substantial PFS benefit in both MMRd (HR 0.30) and pMMR (HR 0.54) cohorts, with 12-month PFS rates of 74% versus 38% in the MMRd group [123,124]. Furthermore, the first-line randomized phase III DUO-E trial showed that adding durvalumab to chemotherapy, followed by durvalumab maintenance with or without olaparib, improved PFS. Notably, the durvalumab plus olaparib arm achieved a median PFS of 15.1 months compared with 9.6 months in the control arm (HR 0.55) [5,114,125]. However, this result should be interpreted with caution: the two durvalumab-containing arms were each compared with the control arm rather than with each other, and overall survival data at the interim analysis were immature (28% maturity; median follow-up 18.7 months) [114,126]. Moreover, in RUBY Part 2, adding niraparib to dostarlimab maintenance improved PFS but did not improve overall survival (see Section 8.5.4) [126]. Whether PARP inhibition adds clinically meaningful benefit to immunotherapy therefore remains unproven. In addition, the first-line phase III AtTEnd trial confirmed that atezolizumab plus chemotherapy significantly improved PFS in MMRd patients (HR 0.36), although the benefit in the pMMR subgroup was less pronounced [16,28]. In the NCCN Guidelines, carboplatin/paclitaxel plus dostarlimab or pembrolizumab (the latter not for carcinosarcoma) is a category 1 preferred first-line option for stage III–IV and recurrent disease irrespective of MMR status, carboplatin/paclitaxel plus durvalumab is category 1 for dMMR tumors only, and durvalumab plus olaparib for pMMR tumors is category 2B, whereas atezolizumab-based chemo-immunotherapy is not currently listed [106].

#### 8.5.4. Maintenance and Future Strategies

Beyond frontline treatment, maintenance immunotherapy strategies are also under investigation. In RUBY Part 2, first-line carboplatin/paclitaxel plus dostarlimab followed by dostarlimab plus niraparib maintenance improved median PFS compared with placebo plus chemotherapy followed by placebo maintenance in both the overall population (14.5 vs. 8.3 months; HR 0.60, 95% CI 0.43–0.82) and the MMRp/MSS population (14.3 vs. 8.3 months; HR 0.63, 95% CI 0.44–0.91). However, in the full publication, this PFS benefit did not translate into an overall survival benefit: at the second interim analysis (median expected follow-up 36.2 months; 40.9% maturity), OS was not improved in either the overall population (HR 1.2, 95% CI 0.81–1.78) or the MMRp/MSS population (HR 1.2, 95% CI 0.78–1.85), and no further OS analyses are planned. The four-drug regimen was also associated with more grade ≥3 treatment-related adverse events (70.7% vs. 37.5%) and more discontinuations due to adverse events (38.7% vs. 11.5%). Because the control arm did not receive dostarlimab, the specific contribution of niraparib cannot be determined; nevertheless, given the added toxicity and the absence of a survival benefit, the investigators concluded that their data do not support adding a PARP inhibitor to immune checkpoint inhibition in this population [126]. This regimen is not included in the NCCN Guidelines.

At the same time, immunotherapy is increasingly being investigated in earlier disease settings. Ongoing trials such as RAINBO (MMRd-GREEN) are evaluating adjuvant ICI therapy after surgery in patients with MMRd tumors, guided by molecular classification, while neoadjuvant applications are being explored in localized MMRd tumors to assess the feasibility of surgical de-escalation [127]. Both approaches remain investigational and are not yet part of routine practice. Collectively, these studies suggest that immunotherapy is expanding beyond the recurrent setting and is becoming an integral component of personalized treatment strategies across the continuum of EC management.

#### 8.5.5. Management of Uterine Carcinosarcoma

According to the NCCN guidelines, a major difference in the management of UCS and endometrioid endometrial carcinoma lies in the need for postoperative adjuvant therapy. Patients with early-stage endometrioid carcinoma without high- or high-intermediate-risk features may undergo observation, whereas those at higher risk may receive vaginal brachytherapy or other adjuvant treatments according to their individual risk profiles. In contrast, systemic chemotherapy is generally considered even for early-stage UCS, with carboplatin plus paclitaxel as the preferred regimen. Vaginal brachytherapy or external beam radiotherapy may also be incorporated according to disease stage and recurrence risk [106].

For advanced, metastatic, or inoperable UCS, carboplatin plus paclitaxel remains a backbone of first-line systemic therapy. Surgery may be considered upfront when complete macroscopic cytoreduction is deemed achievable. Compared with low risk endometrioid carcinoma, fewer patients with UCS are suitable for observation alone, reflecting its greater propensity for recurrence and distant metastasis [106].

### 8.6. Molecularly Guided Therapy Design and Implementation

The prognostic gradients described in Section 5.3 have already changed therapy design. In the adjuvant setting, treatment was traditionally assigned according to clinicopathological risk groups; the ESGO–ESTRO–ESP guidelines now integrate molecular class into these risk groups and recommend molecular classification for all ECs [128]. Long-term data support this shift. In PORTEC-3, the benefit of adding chemotherapy to radiotherapy was concentrated in p53abn tumors (10-year OS 52.7% vs. 36.6%), with no significant benefit in POLEmut or MMRd tumors [99]. Molecular class also predicted response to radiotherapy in PORTEC-1 and PORTEC-2 [89]. PORTEC-4a then provided randomized evidence that molecular profile-based allocation is feasible: 46% of patients in the molecular profile arm had a favorable profile and were spared adjuvant treatment, and the 5-year vaginal recurrence rate remained within the non-inferiority margin (4.5% vs. 1.6% with standard brachytherapy) [37]. In advanced or recurrent disease, MMR status determines the magnitude of benefit from first-line chemo-immunotherapy (Section 8.5.3) [121,122,123,124], and HER2 and ER status identify candidates for HER2-directed and endocrine-based regimens (Section 8.3 and Section 8.4) [105,108,109]. The level of evidence, treatment line, molecular subgroup, and NCCN status of selected systemic therapies for advanced or recurrent EC are summarized in Table 4. Because each of these decisions depends on a molecular result, molecular classification should no longer be regarded as optional or reserved for selected high-risk cases. It should be performed routinely at diagnosis, ideally on the preoperative biopsy, where it can also inform surgical planning [10,96,97].

Looking ahead, molecular class could serve as the backbone of personalized treatment algorithms in which each subgroup follows a distinct therapeutic strategy. This approach is being tested prospectively in the RAINBO program, which evaluates treatment de-escalation in POLEmut tumors, chemoradiotherapy with or without olaparib in p53abn tumors, the addition of durvalumab to radiotherapy in MMRd tumors, and progestin combined with radiotherapy instead of chemoradiotherapy in ER-positive NSMP tumors [127]. Beyond the four molecular classes, additional markers may further individualize treatment intensity, including HER2 in p53abn tumors; ER, L1CAM, and CTNNB1 status in NSMP tumors; and circulating tumor DNA-based detection of minimal residual disease (see Section 9) [93]. Implementation will require four elements: (i) reflex molecular testing on the diagnostic biopsy, with an integrated histomolecular report available before treatment planning; (ii) low-cost and rapid assays, such as MMR and p53 IHC combined with targeted POLE testing, to ensure access in resource-limited settings [7,9]; (iii) review of results by a multidisciplinary tumor board, particularly for multiple-classifier tumors and discordant results; and (iv) prospective registries and biomarker-stratified trials to confirm that molecularly guided escalation and de-escalation improve survival, or at least do not compromise it.

## 9. Discussion

This review integrates histopathological, genomic, signaling pathway, and therapeutic evidence in EC, with particular emphasis on how molecular biology can inform clinical management. Molecular classification has substantially improved risk stratification and treatment selection, but classification alone does not fully explain therapeutic response. A major strength of mechanistic studies is therefore that they provide a biological framework for understanding why tumors within the same molecular class or stage may respond differently and why rational combination strategies may be required. For example, interaction between estrogen receptor and PI3K/AKT/mTOR signaling, together with persistent activation of the PI3K/AKT/mTOR pathway in progestin-resistant tumors, provides a rationale for combining endocrine therapy with everolimus. PI3K pathway activation has also been implicated in acquired resistance to CDK4/6 inhibition [105,110]. Similarly, the dependence of p53-deficient tumors on the G2/M checkpoint provides a rationale for Wee1 inhibition [38], while reciprocal activation of the MAPK and PI3K/AKT/mTOR pathways may explain why blockade of a single pathway is insufficient in some tumors [59,60]. Wnt/β-catenin activation in CTNNB1-mutant NSMP tumors and the immunosuppressive microenvironment associated with p53abn tumors may likewise contribute to resistance to immunotherapy. These observations connect the molecular biology of EC with therapeutic development, but they should not be interpreted as established treatment-selection criteria. Most remain hypothesis-generating, and only a subset has been evaluated prospectively in clinical trials. The experience with selinexor illustrates this distinction: although restoration of nuclear retention of wild-type p53 provides a plausible biological rationale, the reported benefit in TP53 wild-type tumors was derived from a subgroup analysis without adjustment for multiplicity.

The limitations of current molecular classification become particularly apparent in tumors that do not fit neatly into a single therapeutic category. Multiple-classifier tumors are an important example. Although hierarchical assignment provides a practical method of classification, it does not necessarily resolve the therapeutic implications of coexisting molecular alterations. For MMRd–p53abn tumors, for instance, it remains uncertain whether the MMRd phenotype predicts the same magnitude of benefit from immune checkpoint inhibitors (ICIs) as in MMRd tumors without p53 abnormality, or whether the adverse features associated with p53abn disease justify treatment intensification. A similar uncertainty exists for advanced POLEmut disease. The excellent prognosis of early-stage POLEmut tumors has supported interest in treatment de-escalation, but these data cannot simply be extrapolated to stage III–IV disease or to POLEmut tumors with a coexisting p53abn pattern. Accordingly, the ESGO–ESTRO–ESP guidelines provide no firm recommendation for these situations and allow individualized de-escalation only after multidisciplinary assessment [128]. These examples illustrate a broader limitation of hierarchical classification: assigning a tumor to one molecular group is useful for risk stratification but may obscure biologically relevant heterogeneity within that group.

This intragroup heterogeneity is also evident in treatment response and highlights the need to move from prognostic molecular classification toward predictive biomarker refinement. The clearest example is MMRd disease. Although MMR status identifies a population with substantial sensitivity to ICIs, not all MMRd tumors respond, and both primary and acquired resistance occur. Therefore, the next step is not simply to identify additional molecular classes, but to determine which biological features within established classes explain treatment sensitivity or resistance. Candidate predictors of ICI benefit include alterations in antigen-presentation genes, interferon-γ signatures, clonal tumor mutational burden, and the density and spatial distribution of CD8+ T cells. Tissue and blood specimens collected in large, randomized trials such as RUBY, NRG-GY018, and DUO-E provide an opportunity to test whether these biomarkers add predictive information beyond MMR status. In parallel, circulating tumor DNA (ctDNA)-based detection of minimal residual disease may complement static molecular classification by providing a dynamic measure of residual disease and recurrence risk. Such an approach could eventually support escalation or de-escalation of adjuvant treatment, particularly in MMRd and p53abn tumors, but prospective interventional validation is required before ctDNA-guided treatment can be incorporated into routine practice.

The same principle applies to targeted therapy: the presence of a potentially actionable pathway does not by itself establish that inhibition of that pathway will produce clinical benefit. Biomarkers must therefore be developed together with the therapies they are intended to guide. Homologous recombination deficiency and BRCA status, for example, are biologically plausible markers for PARP inhibitor sensitivity, but their predictive value in EC remains uncertain. Similarly, the development of antibody–drug conjugates require standardized, EC-specific assessment of target expression, including HER2, TROP2, folate receptor-α, and B7-H4, to distinguish target expression from true treatment sensitivity. Within the heterogeneous NSMP group, integration of CTNNB1 and L1CAM status with ER expression and tumor grade may provide more informative risk stratification than the NSMP designation alone. Markers reflecting pathway activity, including PTEN loss, PIK3CA mutation, and CTNNB1 exon 3 mutation, may also help identify patients for pathway-directed combinations. However, these markers should be considered candidate predictive biomarkers rather than clinical selection criteria until their value is demonstrated prospectively.

These uncertainties have direct implications for the design of future clinical trials. If molecular heterogeneity is the major limitation of current classification, future studies should be designed to test treatments within biologically defined populations rather than relying predominantly on histology and stage. Biomarker-stratified and umbrella trials, with molecular class incorporated prospectively into study design, provide one approach, as illustrated by the RAINBO program [127]. For favorable molecular groups, adequately powered non-inferiority trials are needed to determine whether treatment can be safely de-escalated without compromising disease control. This is particularly important for POLEmut disease, where the strong rationale for de-escalation in early-stage tumors contrasts with the limited evidence in stage III–IV disease. Conversely, pathway-directed combination trials should focus on patients selected according to the biological mechanism being targeted, rather than evaluating combinations in unselected EC populations. Examples include endocrine therapy combined with PI3K/AKT/mTOR or CDK4/6 inhibition and dual PI3K–MAPK blockade. Multiple-classifier tumors and rare histological subtypes should also be deliberately incorporated into prospective trials or registries so that their management is informed by direct evidence rather than extrapolation from dominant molecular groups.

Finally, the therapeutic questions facing EC are changing as ICIs move into first-line treatment. The major question is no longer only which patients should receive immunotherapy, but how patients should be treated after progression on an ICI-containing regimen. Prospective sequencing studies are therefore needed to determine whether post-ICI therapy should be selected according to molecular subtype, the mechanism of resistance, or additional biomarkers. Other unresolved questions, including the optimal duration of ICI therapy and the role of neoadjuvant immunotherapy in MMRd tumors, should likewise be addressed prospectively. Importantly, promising signals from retrospective or exploratory subgroup analyses should not be treated as definitive evidence of predictive benefit; where they may alter clinical practice, confirmation in adequately powered prospective trials is required. Taken together, the next phase of precision medicine in EC should move beyond assigning tumors to molecular classes toward explaining heterogeneity within those classes. Integrating molecular classification with validated predictive biomarkers, dynamic measures of residual disease, and mechanism-based clinical trials will be essential to translate increasingly detailed biological knowledge into more precise treatment selection and clinically meaningful benefit.

## 10. Conclusions

The management of EC has undergone a fundamental paradigm shift, from a traditional histopathological framework toward a biology-driven model based on molecular characterization. In parallel, growing knowledge of key oncogenic pathways, including the PI3K/AKT/mTOR, Wnt/β-catenin, and MAPK signaling networks, has supported the development of targeted therapies, and emerging agents such as ADCs, PARP inhibitors, and other molecularly targeted drugs continue to broaden the options for advanced and recurrent disease. Looking ahead, the future of EC management lies in integrating molecular classification, advanced diagnostics, and precision therapeutics. Ongoing studies aim to refine risk stratification beyond the current TCGA framework and to optimize molecularly guided treatment strategies further.

## Figures and Tables

**Table 1:** Histopathological Characteristics of Major Endometrial Cancer Subtypes.

Histologic Subtype	Incidence	Histopathological Features	Molecular Features	Clinical Behavior	Reference
Endometrioid Endometrial Carcinoma (EEC)	>80%	Glandular/villoglandular architecture; graded as low-grade (G1–2) or high-grade (G3).	Frequently ER/PR positive, High-grade tumors frequently associated with MMRd or POLE mutations.	Most common subtype and generally estrogen dependent.	[5,6,13,14,15]
Serous Carcinoma (SEC)	10%	Papillary architecture with marked nuclear atypia and high mitotic activity.	TP53 mutations with aberrant p53 expression.	Highly aggressive; responsible for 40% of EC-related deaths.	[5,12,16]
Clear Cell Carcinoma (CCC)	<5%	Clear cytoplasm and hobnail cells; tubulocystic, papillary, or solid patterns.	Typically ER/PR negative.	Aggressive behavior and relative chemoresistance.	[5,8,12]
Carcinosarcoma (CS)	<5%	Biphasic tumor with epithelial and mesenchymal components.	Frequent TP53 mutations.	Extremely aggressive with high recurrence and poor prognosis.	[5,11,17,18,19]
Undifferentiated/Dedifferentiated Carcinoma (UDC/DDC)	Rare	Undifferentiated cells with absent or minimal epithelial differentiation.	SWI/SNF complex loss; frequent MMR deficiency.	Highly aggressive and associated with poor outcomes.	[5,12,14,20]

Abbreviations: EC, endometrial cancer; ER, estrogen receptor; PR, progesterone receptor; MMRd, mismatch repair deficiency.

**Table 2:** Major Genetic Alterations and Genomic Features in Endometrial Cancer.

Genetic	Frequency	Function	Mechanism	Clinical Relevance
PTEN	40–80% ^a^	Tumor suppressor; negative regulator of PI3K/AKT signaling.	Causes constitutive PI3K/AKT activation, promoting proliferation.	Early event in endometrioid EC; frequently detected in AEH.
PIK3CA	40–50% ^b^	Encodes PI3K catalytic subunit (p110α).	Gain-of-function mutations increase PI3K activity.	Frequently coexists with PTEN loss, especially in endometrioid subtypes.
PIK3R1	Common in endometrioid EC ^a^	Encodes PI3K regulatory subunit (p85α).	In-frame insertions/deletions enhance AKT phosphorylation.	
TP53	>90% of serous carcinoma ^a^ and carcinosarcoma ^d^	Tumor suppressor regulating DNA repair, apoptosis, and cell cycle.	Mutation causes genomic instability, aneuploidy, and copy-number alterations.	Hallmark of serous-like/p53-abnormal EC; associated with aggressive behavior and poor prognosis.
ARID1A	30–50% ^a^	SWI/SNF chromatin remodeling complex component.	Loss-of-function impair chromatin remodeling and DNA repair.	Promotes progression from AEH to carcinoma.
KRAS	10–30% ^a^	MAPK/ERK pathway regulator.	Activating mutations stimulate proliferation and survival.	Early event in tumorigenesis; associated with mucinous differentiation.
CCNE1	Common in CNH tumors ^c^	Encodes Cyclin E1, a key regulatory protein in cell cycle.	Promotes G1/S transition and replication stress.	Associated with aggressive disease and chemoresistance.
ERBB2	25% of CNH EC ^c^; 17–33% of carcinosarcoma ^d^	Encodes HER2, a transmembrane TKR.	Amplification enhances proliferative signaling.	Biomarker for HER2-targeted therapy; strong association with TP53 mutation in 94–97% of cases.

Abbreviations: EC, endometrial cancer; AEH, atypical endometrial hyperplasia; CNH, copy-number high; HER2, human epidermal growth factor receptor 2; TKR, tyrosine kinase receptor. Frequencies are approximate ranges compiled from the cited studies; they vary with the histotyping composition of the cohort and the assay used and are intended to illustrate relative prevalence rather than precise population estimates. Source population and testing method: ^a^ TCGA cohort of endometrioid and serous carcinomas, whole-exome sequencing [15]; ^b^ unselected EC cohorts of all histotypes, tumor sequencing pooled in a meta-analysis [23]; ^c^ copy-number-high EC, SNP-array (TCGA) or copy-number signature analysis [15,24]; ^d^ carcinosarcoma and HER2-amplified EC series, whole-exome sequencing, immunohistochemistry/in situ hybridization, or NGS [17,22,25].

**Table 3:** Molecular Classification and Features of Endometrial Cancer According to TCGA and ProMisE.

TCGA	Frequency	Features	Prognosis	ProMisE
POLE ultramutated	7–10%	Mutations in the POLE exonuclease domain; Extremely high TMB; Frequently FIGO grade 3 EC; Younger age, lower BMI.	Excellent with very low recurrence risk.	POLEmut
MSI hypermutated	28–30%	MMRd caused by MLH1 promoter hypermethylation or Lynch syndrome; High MSI; TMB roughly 10-fold higher than the background; Predominantly endometrioid tumors with prominent TILS.	Intermediate.	MMRd
Copy-number low	38–40%	Low mutation burden; Low SCNAs; Frequently hormone receptor-positive; Mostly low-grade EC.	Generally favorable; CTNNB1-mutation with increased recurrence risk.	NSMP (p53wt)
Copy-number high	26%	Low mutation burden; Extensive SCNAs; TP53 mutations in ~90–95% of cases; Mainly serous carcinomas, or high-grade endometrioid carcinomas and carcinosarcomas.	Worst prognosis with aggressive clinical behavior.	p53abn

Abbreviations: EC, endometrial cancer; TMB, tumor mutational burden; MMRd, mismatch repair deficiency; MSI, microsatellite instability; TILS, tumor-infiltrating lymphocytes; SCNA, somatic copy number alteration.

**Table 4:** Level of evidence, treatment line, molecular subgroup, and NCCN status of selected systemic therapies for advanced or recurrent endometrial cancer.

Regimen	Trial (Design)	Setting/Line	Molecular Subgroup	Key Efficacy	Status *
*Randomized phase III evidence*					
Dostarlimab + carboplatin/paclitaxel	RUBY Part 1 (phase III)	First-line; primary stage III–IV or first recurrence	MMRd; pMMR	PFS HR 0.28 (MMRd); HR 0.76 (pMMR)	NCCN category 1, preferred
Pembrolizumab + carboplatin/paclitaxel	NRG-GY018 (phase III)	First-line; primary stage III–IV or recurrence	MMRd and pMMR cohorts	PFS HR 0.30 (MMRd); HR 0.54 (pMMR)	NCCN category 1, preferred (not for carcinosarcoma)
Durvalumab + carboplatin/paclitaxel, then durvalumab ± olaparib	DUO-E (phase III)	First-line and maintenance	MMRd; pMMR	PFS 15.1 vs. 9.6 months, HR 0.55 (durvalumab + olaparib vs. control)	Durvalumab: category 1 (MMRd only); + Olaparib: category 2B (pMMR)
Atezolizumab + carboplatin/paclitaxel	AtTEnd (phase III)	First-line; advanced or recurrent	MMRd; pMMR	PFS HR 0.36 (MMRd); less pronounced in pMMR	Not listed in NCCN
Lenvatinib + pembrolizumab	KEYNOTE-775/Study 309 (phase III)	After ≥1 platinum-based regimen; ICI-naïve	All-comers; pMMR	OS 18.0 vs. 12.2 months (pMMR)	NCCN category 1 (previously treated pMMR)
Selinexor maintenance	SIENDO (phase III, placebo-controlled)	Maintenance after platinum-based chemotherapy	TP53wt (exploratory subgroup)	PFS 28.4 vs. 5.2 months, HR 0.41 (TP53wt)	Investigational; not in NCCN
Dostarlimab + chemotherapy, then dostarlimab + niraparib	RUBY Part 2 (phase III)	First-line and maintenance	Overall; pMMR	PFS 14.3 vs. 8.3 months (pMMR; HR 0.63); no OS benefit (HR 1.2)	Not supported by investigators; not in NCCN
*Randomized phase II evidence*					
Everolimus + letrozole	GOG-3007 (noncomparative randomized phase II)	≤1 prior chemotherapy regimen	Not molecularly selected	Median PFS 28 months (chemotherapy-naïve) vs. 4 months (prior chemotherapy)	NCCN preferred hormonal option
Palbociclib + letrozole	PALEO (randomized phase II, placebo-controlled)	Mostly second line or later (after≥1 prior systemic therapy)	ER-positive endometrioid	PFS 8.3 vs. 3.0 months, HR 0.56	NCCN: useful in certain circumstances
Carboplatin/paclitaxel + trastuzumab	Fader et al. (randomized phase II)	First line; primary stage III–IV or recurrent	HER2-positive serous carcinoma	PFS 12.6 vs. 8.0 months, HR 0.44	NCCN preferred (HER2-positive serous carcinoma/carcinosarcoma)
Vistusertib + anastrozole	VICTORIA (randomized phase I/II)	Recurrent or metastatic	Hormone receptor-positive	Median PFS 5.2 months; ORR 24.5%	Investigational
Olaparib maintenance	UTOLA (randomized phase IIb, placebo-controlled)	Maintenance after platinum-based chemotherapy	HRD-positive (subgroup)	PFS 5.4 vs. 3.6 months, HR 0.59	Olaparib monotherapy not recommended
Bevacizumab + chemotherapy	GOG-86P (randomized phase II)	First-line	TP53-mutant (post hoc)	Greater benefit suggested in TP53-mutant tumors	Molecular association hypothesis-generating
*Single-arm phase I/II evidence*					
Pembrolizumab	KEYNOTE-158 (phase II basket)	Previously treated	MSI-H/MMRd	ORR 48–57.1%	NCCN biomarker-directed option
Dostarlimab	GARNET (phase I)	After platinum-based chemotherapy	MMRd (pMMR ORR 13.4–15.4%)	ORR 42.3–45.5%	NCCN biomarker-directed option
Nivolumab, durvalumab, avelumab	Phase II studies (e.g., PHAEDRA)	Previously treated	MMRd	ORR 23–46%	Avelumab and nivolumab listed for MSI-H/MMRd
Trastuzumab deruxtecan	DESTINY-PanTumor02; STATICE (phase II)	Previously treated	HER2 IHC 2+/3+ EC; HER2-expressing carcinosarcoma	ORR 57.5% (EC); 54.5%/70% (HER2-high/-low carcinosarcoma)	NCCN biomarker-directed (HER2 IHC 2+/3+ EC; carcinosarcoma IHC 1+~3+)
Abemaciclib + letrozole	Single-arm Phase II	Recurrent	ER-positive	ORR 30%; median PFS 9.1 months	NCCN: useful in certain circumstances
Sacituzumab govitecan	TROPiCS-03 (phase II basket); ASCENT-GYN-01 (phase III, ongoing)	Previously treated (after platinum and anti PD-(L)1 therapy)	Not selected	ORR 22%	Investigational; not in NCCN
Adavosertib	Single-arm Phase II	Recurrent	Serous carcinoma (largely p53abn)	Responses in ~30%	Investigational; not in NCCN

* Guideline status refers to the NCCN Clinical Practice Guidelines in Oncology: Uterine Neoplasms, version 2.2026 [106]. Efficacy data are those reported in Section 8.4 and Section 8.5; results of subgroup, exploratory, or post hoc analyses are hypothesis-generating. Abbreviations: MMRd, mismatch repair-deficient; ER, estrogen receptor; HR, hazard ratio; HRD, homologous recombination deficiency; ICI, immune checkpoint inhibitor; IHC, immunohistochemistry; MSI-H, microsatellite instability-high; ORR, objective response rate; OS, overall survival; p53abn, p53-abnormal; PFS, progression-free survival; pMMR, mismatch repair-proficient; TP53wt, TP53 wild-type.

## Data Availability

No new data were created or analyzed in this study. Data sharing is not applicable to this article.
